# Cell surface CD55 traffics to the nucleus leading to cisplatin resistance and stemness by inducing PRC2 and H3K27 trimethylation on chromatin in ovarian cancer

**DOI:** 10.1186/s12943-024-02028-5

**Published:** 2024-06-10

**Authors:** Rashmi Bharti, Goutam Dey, Debjit Khan, Alex Myers, Olivia G. Huffman, Caner Saygin, Chad Braley, Elliott Richards, Naseer Sangwan, Belinda Willard, Justin D. Lathia, Paul L. Fox, Feng Lin, Babal Kant Jha, J. Mark Brown, Jennifer S. Yu, Mohammed Dwidar, Amy Joehlin-Price, Roberto Vargas, Chad M. Michener, Michelle S. Longworth, Ofer Reizes

**Affiliations:** 1 Department of Cardiovascular and Metabolic Sciences, Lerner Research Institute, Cleveland Clinic Foundation, 9500 Euclid Avenue Cleveland Clinic, Cleveland, OH 44195 USA; 2 Reproductive, Endocrinology, and Infertility, Obstetrics and Gynecology Institute, Cleveland Clinic Foundation, Cleveland, OH USA; 3 Microbiome Analytics and Composition Core Facility, Lerner Research Institute, Cleveland Clinic Foundation, Cleveland, OH USA; 4 Case Comprehensive Cancer Center, Cleveland, OH USA; 5 Proteomics and Metabolomics Core, Lerner Research Institute, Cleveland Clinic, Cleveland, OH 44106 USA; 6 Department of Immunity and Inflammation, Lerner Research Institute, Cleveland Clinic, Cleveland, OH USA; 7 Center for Immunotherapy & Precision Immuno-oncology, Lerner Research Institute, Cleveland Clinic Foundation, Cleveland, OH USA; 8 Department of Cancer Biology, Lerner Research Institute of the Cleveland Clinic, Cleveland, OH USA; 9 Microbial Culturing and Engineering Facility, Cleveland Clinic, Cleveland, OH USA; 10 Anatomic Pathology, Pathology and Lab Medicine Institute, Cleveland Clinic Foundation, Cleveland, OH USA; 11 Department of Gynecologic Oncology, Obstetrics and Gynecologic Institute, Cleveland Clinic Foundation, Cleveland, OH USA; 12 Present address: Section of Hematology/Oncology, Department of Medicine, University of Chicago, Chicago, IL USA

## Abstract

**Background:**

Platinum resistance is the primary cause of poor survival in ovarian cancer (OC) patients. Targeted therapies and biomarkers of chemoresistance are critical for the treatment of OC patients. Our previous studies identified cell surface CD55, a member of the complement regulatory proteins, drives chemoresistance and maintenance of cancer stem cells (CSCs). CSCs are implicated in tumor recurrence and metastasis in multiple cancers.

**Methods:**

Protein localization assays including immunofluorescence and subcellular fractionation were used to identify CD55 at the cell surface and nucleus of cancer cells. Protein half-life determinations were used to compare cell surface and nuclear CD55 stability. CD55 deletion mutants were generated and introduced into cancer cells to identify the nuclear trafficking code, cisplatin sensitivity, and stem cell frequency that were assayed using in vitro and in vivo models. Detection of CD55 binding proteins was analyzed by immunoprecipitation followed by mass spectrometry. Target pathways activated by CD55 were identified by RNA sequencing.

**Results:**

CD55 localizes to the nucleus of a subset of OC specimens, ascites from chemoresistant patients, and enriched in chemoresistant OC cells. We determined that nuclear CD55 is glycosylated and derived from the cell surface pool of CD55. Nuclear localization is driven by a trafficking code containing the serine/threonine (S/T) domain of CD55. Nuclear CD55 is necessary for cisplatin resistance, stemness, and cell proliferation in OC cells. CD55 S/T domain is necessary for nuclear entry and inducing chemoresistance to cisplatin in both in vitro and in vivo models. Deletion of the CD55 S/T domain is sufficient to sensitize chemoresistant OC cells to cisplatin. In the nucleus, CD55 binds and attenuates the epigenetic regulator and tumor suppressor ZMYND8 with a parallel increase in H3K27 trimethylation and members of the Polycomb Repressive Complex 2.

**Conclusions:**

For the first time, we show CD55 localizes to the nucleus in OC and promotes CSC and chemoresistance. Our studies identify a therapeutic mechanism for treating platinum resistant ovarian cancer by blocking CD55 nuclear entry.

**Supplementary Information:**

The online version contains supplementary material available at 10.1186/s12943-024-02028-5.

## Introduction

Epithelial ovarian cancer (EOC) is the second most common gynecologic malignancy in the USA. In 2023, 19,710 women received a diagnosis of ovarian cancer while approximately 13,270 women succumbed to their disease in the US [1]. Tumor debulking followed by platinum therapy is the standard of care in the treatment of EOC. Despite the initial response, tumor recurrence and cisplatin resistance underlie the poor overall survival. We and others have shown that resident cancer stem cells (CSCs) are significantly enriched in EOC, a population thought to underlie tumor recurrence, chemoresistance, and metastasis [2]. Therapeutics to target CSCs are in development and undergoing evaluation in the clinic, thus warranting additional targets for drug development. Our previous studies identified that the membrane complement regulatory protein CD55 induces CSCs and increases chemoresistance in endometrioid ovarian cancer [3].

CD55 or decay accelerating factor (DAF) is a cell surface glycophosphatidylinositol (GPI) anchored protein that participates in the regulation of complement activation and protects cells from complement-mediated lysis [4]. Structurally, CD55 has three major domains including four short consensus repeats called SCR-1, SCR-2, SCR-3, SCR-4, a Serine/Threonine (S/T) rich domain, and a GPI anchor domain [5]. Functionally, the SCR domains attenuate complement activation, while the GPI domain tethers CD55 to the outer surface of the cell membrane in lipid microdomains. The S/T domain is glycosylated by O-linkage, but its role remains unclear.

We previously found that CD55 promotes CSCs by activating ROR2/JNK signaling and upregulating SOX2, Nanog, and OCT4 expression in endometrioid ovarian cancer [3]. In parallel, CD55 also induces DNA repair genes via activation of LCK signaling in ovarian cancer cells [3]. Subsequent studies found CD55 is amplified in breast, colon, gastric, lung, renal, thyroid, brain, cervical, and ovarian cancers [6]. Recent studies show that cancer cells utilize CD55 to promote cell proliferation and survival [7]. Mechanistically, CD55 accelerates tumor growth by activating oncogenic pathways including JNK, LCK/FYN, JAK/STAT3, and NF-kB/MAPK in several cancers [3, 7–9]. Ovarian CSCs express high levels of CD55 and shRNA mediated silencing in ovarian cancer cells leading to attenuation of self-renewal and increased cisplatin sensitivity [3]. Collectively, the studies indicate CD55 is a unique therapeutic target, however, selective targeting is challenging as CD55 is essential in attenuating complement-mediated cell lysis and physiologic functions.

Here, we identify CD55 as a nuclear resident protein in chemoresistant ovarian cancer cells and patient tumor specimens that can associate with chromatin. We define a unique nuclear trafficking code in CD55, specifically the S/T domain, that is sufficient to induce CSC self-renewal and chemoresistance as well as binding to chromatin. Nuclear CD55 (nCD55) interacts with and suppresses the protein expression of the epigenetic regulator ZMYND8 leading to increased tumorigenesis. The findings unmask a new function for CD55 in the nucleus and offer a selective targeting approach as nCD55 is solely found in cancer cells.

## Materials and methods

### Cell line and culture conditions

Cancer cells and others, CP70 (Culture media: DMEM 10% FBS), A2780 (Culture media DMEM: 10% FBS), SKOV3 (Culture media: McCoy5a 10% FBS), TOV112D (Culture media: MCDB 15% FBS), OVCAR8 (Culture media: DMEM 10% FBS), OV81 (Culture media: DMEM 10% FBS), HEK293T (Culture media: DMEM 10% FBS), Jurkat (Culture media: RPMI 10% FBS) cells were grown in recommended growth media in humidified incubator containing 5% CO_2_. For each experiment, cells were grown up to a confluence of ~ 70% and treated or analyzed as indicated.

### Chemicals and reagents

Cell culture medias were purchased from the Lerner Research Institute Media Core at the Cleveland Clinic. Cisplatin was purchased from the Cleveland Clinic Pharmacy. Cell titer glow reagent (Promega), FBS (Atlas Biologicals), anti-CD55 antibody from Proteintech and EMD Millipore, anti-ZMYND antibody (Proteintech), anti-GAPDH antibody (Proteintech), anti-Lamin A/C antibody (Proteintech), anti-α Tubulin antibody (Proteintech), Goat anti-Rabbit IgG Alexa Fluor 488 (Thermo Scientific), and Goat anti-mouse IgG Alexa Fluor 568 (Thermo Scientific) were used in different studies. Details of chemicals/reagents described in supplementary Table 1.

### Generation of wild type CD55 and domain deletion mutant lentiviral plasmids

Wild type CD55 and deletion mutants (Δ1, Δ2, Δ3, Δ4, Δ1234, ΔS/T, Δ34, and Δ34S/T) were generated using D-TOPO cloning method. Briefly, the gene block of CD55 cDNA was purchased from Integrated DNA Technologies (IDT). CD55 cDNA was cloned into pENTR™/D-TOPO® Vector followed by LR recombination reaction to clone CD55 into the destination vector pLenti CMV Puro DEST. pENTR™/D-TOPO® CD55 mutants were generated using primers for site-directed mutagenesis (Supplementary Table 5). Mutants were cloned into the destination vector pLenti CMV Puro DEST for mammalian expression. Details of the plasmid sequence of wild-type CD55 and domain deletion CD55 mutants described in supplementary Table 4.

### Generation of CRISPR/Cas9 knockout cells

Gene knock outs were performed from indicated cancer cells using a CD55 CRISPR/Cas9 knockout plasmid (Santa Cruz Biotechnology) or ZMYND8 CRISPR/Cas9 knockout plasmid (Santa Cruz Biotechnology). Briefly, cancer cells were seeded in six well plates and transfected with GFP labelled CRISPR/CAS9 knockout plasmids using Lipofectamine 3000. After 24 h, the transfection media was replaced with fresh serum-enriched medium. Cells were sorted by flow cytometry and GFP positive cells were collected as single cells in 96 well plates and cultured for 10 days. Each clone was screened to check for knockout efficiency. After confirming sufficient knockout, cells were grown and used in the indicated experiments.

### Limiting dilution assays to determine cancer stem cell frequency

Ovarian cancer cells were counted and plated as single cells in nonadherent 96-well plates in 200 µL of stem cell media. Cells were plated at a density of 1, 5, 10, and 20 cells per well in triplicate rows. Stem cell media contained serum free DMEM/F-12, Fibroblast growth factor (bFGF) 20 ng/mL, Epidermal growth factor (EGF) 10ng/mL, B27 supplement 2%, and Insulin 10 µg/mL. After two weeks, each well was examined under a phase contrast microscope to detect tumorsphere-formation. Stem cell frequency or sphere-forming frequency was estimated using ELDA (Extreme limiting dilution algorithm) software (http://bioinf.wehi.edu.au/software/elda/) [10].

### Cell proliferation assay using IncuCyte

Cancer cell proliferation was measured using an earlier reported method [11]. Briefly, CP70 cells (CD55 OE, KO, Δ1, Δ2, Δ3, Δ4, Δ1234, ΔS/T, Δ34, and Δ34S/T) were collected from cells during growing phase. Cells were seeded (1000 cells/well) in Geltrex-coated 96-well plates and incubated in the IncuCyte Live Cell Analysis System (Sartorius). Cell proliferation was measured up to 96 h as cell count normalized to day zero.

### Immunoblot analysis

Immunoblots were performed to check protein expression as previously reported [12]. In brief, at the end of each study, plates were washed 3X with ice-cold PBS and placed on ice. Cells were harvested with NP-40 lysis buffer containing 250 mM NaCl, 50 mM Tris, pH 7.4, 1 mM Na3VO4, 5 mM EDTA, 1% Nonidet™ P40 (NP40), 50 mM NaF, 0.02% NaN3, 2 µg/ml protease cocktail inhibitor and 1mM PMSF by dropwise addition to the plates and kept on ice for few minutes. Cells were scrapped and collected into 1.5 ml centrifuge tube and kept on ice for 1.5 h. Lysates were vortexed vigorously every 10 min. Lysates were then centrifuged for 10 min at 12,000 rpm at 4 °C. The supernatants were collected and placed in a new 1.5 centrifuge tube and kept on ice. Protein concentration was measured by BCA (Thermo Scientific). 6× Laemmli buffer containing β-mercaptoethanol was added to the protein lysates and protein samples were boiled for 6 min. Protein samples were then resolved on SDS-PAGE electrophoresis using precast gel (4–20% Gradient gel, Biorad) and transferred to PVDF membrane using a wet transfer method at 4 °C for overnight. After transfer, PVDF membranes were blocked in 5% BSA for one hour at room temperature followed by the addition of primary antibodies at 4 °C overnight with gentle rocking. The next day, membranes were washed three times with 1×TBST (Tris-Buffered Saline, 0.1% Tween® 20) on a platform shaker followed by incubation with HRP-conjugated secondary antibodies for one hour at room temperature. Membranes were then washed three times with 1×TBST. Immunodetection was carried out using a chemiluminescence reagent (PerkinElmer) in a Chemidoc imaging system (GE Healthcare) and band densitometry was quantified by Image J software.

### Cytoplasmic and nuclear protein fractionation

Cytoplamic and nuclear fractions were isolated using a commercially available NE-PER™ Nuclear and Cytoplasmic Extraction Kit (Thermo Scientific). Briefly, cells were cultured in 100 mm Petri Dish and grown to. ~70% confluency, cells were harvested, and cytoplasmic and nuclear fractions were isolated. After protein isolation, immunoblot study was performed according to the above-mentioned methods. For cytoplasmic protein, α-Tubulin antibody was used as loading control and for nuclear protein, Lamin A/C antibody was used as loading control.

### Subcellular fractionation

Subcellular fractionation assay was performed according to the manufacturer’s instructions (Thermo Scientific). Briefly, ovarian cancer cells were grown in 100 mm Petri dish and grown to 80% confluence. Cells were harvested and washed two times with chilled PBS. The cell pellets were stepwise lysed according to the manufacturer’s protocol. In this experiment, cytoplasmic, soluble nuclear, chromatin-bound, and membrane fraction proteins were isolated to detect. Subcellular fractions were validated using tubulin for cytoplasmic faction, Na+/K + ATPase for cell membrane, Lamin A/C for soluble nuclear, and Histone H3 for chromatin-bound fraction.

### CellTiter-Glo® assay

Cancer cells (CP70 cells 2000/well and SKOV3 4000/well) were seeded in 96 well plates (White non-transparent). The next day, cells were treated with increasing doses of cisplatin (0, 0.1, 0.3, 1, 3, 10, 30, and 100µM) for 48 h. Subsequently, CellTiter-Glo reagent mix (100 µl) was added to each well by replacing 100 µl cisplatin containing media according to the manufacturer’s protocol (Promega). Plates were placed on a platform shaker for 5 min with gentle rocking. The luminescence reading of each well was measured in a luminometer and viability was calculated as a percentage normalized to untreated control. Percent viability for each concentration was plotted using GraphPad Prism software (GraphPad Software, Inc.).

### Cycloheximide treatment and protein stability

Ovarian cancer cells (Parental cells and CD55 WT/mutants transduced cells) were grown in 100 mm Petri dish until 60% confluence. Cells were then treated with cycloheximide for indicated times. Cycloheximide containing media was discarded and washed with chilled PBS two times. Cells were collected by scraping in PBS and centrifuged to obtain cell pellets. Cytoplasmic and nuclear fractions were enriched using commercially available NE-PER™ Nuclear and Cytoplasmic Extraction Reagents (Thermo Scientific). Protein samples were prepared, resolved by SDS-PAGE, and subjected to immunoblot analysis.

### PIPLC treatment in cancer cells

To release CD55 from lipid rafts, Phosphatidylinositol-Specific Phospholipase C (PIPLC, Thermo) was used to cleave the GPI anchor [13]. Briefly, cancer cells were grown in 100 mm Petri dishes to 70% confluence. Fresh media was added to the cells followed by the addition of PIPLC to the media at concentrations of 10, 25, or 35 Unit/ml. Cells were then incubated at 4 °C for 30 min or 37 °C for indicated time with gentle rocking. Cells were then washed with ice-cold D-PBS three times and cytoplasmic and nuclear fractions were isolated. Immunoblots of the enriched fraction were performed to check CD55 expression.

### Analysis of N-linked and O-linked glycosylation of CD55 protein

Cell surface CD55 is heavily O-glycosylated at the serine/threonine rich domain whereas it is lightly N-glycosylated at the SCR1/2 domain [14]. To elucidate whether nuclear CD55 is glycosylated we used a deglycosylation mix II enzyme (NEB) to remove O-linked and N-linked glycosylation. Briefly, cancer cells were grown in 100 mm Petri Dishes to a confluence of 70%, cells were harvested, and cytoplasmic/nuclear fractions prepared. Deglycosylation mix II enzymes were incubated with the nuclear and cytoplasmic fractions according to the manufacturer’s protocol. Protein samples were processed for immunoblot analysis.

For the analysis of N-linked glycosylation of CD55 protein, cancer cells were grown in 100 mm Petri Dishes. We used tunicamycin, a previously reported N-linked glycosylation inhibitor [15]. Cells were grown to 70% confluence and treated with tunicamycin for 24 h. Cells were harvested, and cytoplasmic/nuclear proteins were fractionated. Protein samples were resolved on SDS-PAGE and immunoblotted for CD55.

### Immunofluorescence analyses

Immunofluorescence study was performed to visualize the distribution of CD55 protein in cancer and non-cancerous cells. Briefly, Cells were plated on coverslips in cell media and incubated for 48 h. For processing, cells were washed with D-PBS two times and fixed in a 4% paraformaldehyde solution for 10 min, washed with D-PBS two times. Coverslips were either incubated in 0.01%Triton-X-100 solution for 2–3 min or untreated followed by incubation in blocking buffer (3% BSA + 2% Goat serum in TBST) for 1 h at room temperature. Primary antibodies with indicated dilution were added to the cells and coverslips were incubated in a humidified chamber at 4 °C overnight. The next day, coverslips were washed three times with TBST on a platform shaker. Alexa Flour conjugated secondary antibodies were added to the coverslips and cells were incubated for 1 h at room temperature then washed three times with 1×TBST on a platform shaker. Coverslips were mounted on glass slides using VECTASHIELD mounting media (Vector Lab) containing the DAPI and visualized on Confocal microscope.

### Lentivirus generation and transduction

For the generation of stable overexpression lines, lentivirus particles were used to infect the indicated cells. In brief, 4 × 10^6^ HEK293T cells were seeded in 100 mm Petri Dish. Next, day, HEK293T cells were transfected with pMD2.G (Viral envelope expressing plasmid), pMDLg/pRRE (Packaging plasmid), pRSV-Rev (Packaging plasmid) and pLenti-puro Dest vectors (pLenti CMV Puro DEST, CD55 WT pLenti CMV Puro DEST, Δ1 pLenti CMV Puro DEST, Δ2 pLenti CMV Puro DEST, Δ3 pLenti CMV Puro DEST, Δ4 pLenti CMV Puro DEST, Δ1234 pLenti CMV Puro DEST, ΔS/T pLenti CMV Puro DEST, Δ34S/T pLenti CMV Puro DEST). After 24 h of incubation, fresh DMEM media was added to replace the transfection media and incubated for 24 h. Viral particle-containing media was filtered to remove cell debris and floating cells. In parallel, 0.5 × 10^6^ ovarian cancer cells were seeded in six well plates and viral particle containing condition media was added to the cancer cells. After 24 h, a second batch of fresh viral particles from HEK293T cells was used to replace the first batch of viral-containing media and to infect the cancer cells. Virus transduced cells were kept in the incubator for 48 h and then treated with puromycin to select transduced cells. Once cells were ready, protein expression was evaluated to confirm the efficiency of knockdown or overexpression.

### Immunoprecipitation and co-immunoprecipitation

To determine protein-protein interactions, immunoprecipitation and co-immunoprecipitation studies were performed according to the earlier reported method with few modifications [16]. Briefly, OC cells were lysed with Immunoprecipitation (IP) lysis buffer (Thermo Scientific) supplemented with protease cocktail inhibitor. Alternatively, cytoplasmic or nuclear fractions were isolated and IP lysis buffer was added. For immunoprecipitation, 3 µg of CD55 or ZMYND8 antibodies were added to the protein lysate and incubated for overnight at 4 °C. In parallel, a control antibody (Cell Signaling) was added to the protein lysate. Whole cell, cytoplasmic and nuclear lysates were incubated overnight at 4 °C. The next day, pre-cleaned magnetic A/G bead (Thermo Scientific) was added to the protein lysates containing the antibodies and incubated for 4 h at 4 C with constant rotation. Magnetic beads were collected and washed three times with lysis buffer. Laemmli buffer was added to the beads and boiled for 6 min. Beads were removed to obtain the protein samples and resolved on SDS-PAGE followed by immunoblot analysis.

### CD55 immunoprecipitation and LC-MS/MS

Ovarian cancer cells were grown in 150 mm Petri dishes to 70% confluence. Cells were washed with D-PBS two times and harvested by scrapping in D-PBS. Samples were centrifuged at 2000 rpm for 5 min to collect the cell pellet. Cytoplasmic and nuclear fractions were isolated using NE-PER™ Nuclear and Cytoplasmic Extraction Kit (Thermo). Protein concentration of each fraction was estimated by BCA. CD55 antibody (3 µg) was added to the protein lysates and incubated at 4 °C overnight with continuous and mild rotation. The next day, protein A/G Agarose beads were added to the samples and incubated for 3 h at 4 °C. Agarose beads were washed three times with lysis buffer containing protease cocktail inhibitor. 2x Laemmle buffer was added to the beads and boiled for 5 min. Protein samples were collected and beads were discarded, followed by SDS-PAGE. Intact gels were transferred to the mass-spec core at the Lerner Research Institute (Cleveland Clinic) for LC-MS/MS (Liquid chromatography-tandem mass spectrometry) analysis to identify binding partners of cytoplasmic and nuclear CD55. The gel lanes were analyzed using a GeLC method, where large areas of the gel lane were cut, bands were washed/destained in 50% ethanol, 5% acetic acid, and dehydrated in acetonitrile. The bands were then reduced with DTT and alkylated with iodoacetamide prior to the in-gel digestion. All bands were digested in-gel using trypsin, by adding 5 µL of 10 ng/µL chymotrypsin in 50 mM ammonium bicarbonate and incubating overnight digestion at room temperature to achieve complete digestion. The peptides that were formed were extracted from the polyacrylamide in two aliquots of 30µL 50% acetonitrile with 5% formic acid. These extracts were combined, and half of the protein extracts were evaporated in a Speedvac and resuspended in 30 µL 0.1% formic acid for LCMS analysis.

Samples were analyzed by LC-MS using a Fusion Lumos Tribrid MS (ThermoScientific) equipped with a Dionex Ultimate 3000 nano UHPLC system, and a Dionex (25 cm x 75 μm id) Acclaim Pepmap C18, 2-µm, 100-Å reversed-phase capillary chromatography column. Peptide digests (5 µl) were injected into the reverse phase column and eluted at a flow rate of 0.3 µl/min using mobile phase A (0.1% formic acid in H2O) and B (0.1% formic acid in acetonitrile). The gradient was held at 2%B for 5 min, %B was increased linearly to 35% in 80 min, increased linearly to 90% B in 10 min, and maintained at 90% B for 5 min. The mass spectrometer was operated in a data-dependent manner which involved full scan MS1 (375–1700 Da) acquisition in the Orbitrap MS at a resolution of 120,000. This was followed by CID (1.6 Da isolation window) at 35% CE and ion trap detection. MS/MS spectra were acquired for 3 s. The second method was used for glycopeptide identification and involved full scan MS1 7 (350–1700 Da) acquisition in the Orbitrap MS at a resolution of 120,000. Dynamic exclusion was enabled where ions within 10 ppm were excluded for 60 s.

Raw data were analyzed by using all CID spectra collected in the experiment to search the human SwissProtKB database (downloaded on 4-29-2021, 26,594 entries) and more specifically against the sequence CD55 with the program Sequest which is integrated into Thermo Proteome Discoverer (V2.3) software package. Peptide and protein validation was performed using the percolator node with protein, peptide, and PSM thresholds at < 1% FDR. For the differential enrichment analysis, the protein abundance was estimated using the total number of spectra identified for each protein [17].

### H&E and immunohistochemistry of A2780 CSC tumors

Hematoxylin and eosin (H&E) and immunohistochemistry were utilized in tumor tissues derived from the A2780 CSC mice ovarian tumor model, as described in a previously [3].We analyzed tumors that were enriched for CD55 (A2780 CSC) and two tumors with CD55 silenced [A2780 CSCs CD55 KD1(TRCN0000057167), A2780 CSCs CD55 KD2 (TRCN0000255377)] from our previous publication. H&E and IHC methods were described below.

### Quantitative real-time polymerase chain reaction

Quantitative real-time Polymerase chain reaction (qRT-PCR) was performed according to the earlier reported method [18]. Briefly, cancer cells were grown in 100 mm Petri dishes to 70% confluence. Cells were harvested and total mRNA was separated using an RNA isolation kit (Takara Bio). RNA concentration as well as quality were evaluated using Nanodrop. First strand cDNA synthesis (PrimeScript 1st strand cDNA Synthesis Kit, Takara) was carried out from the isolated RNA. Primers for ZMYND8 and GAPDH were designed using Primer-BLAST (supplementary Table 6). Then we mixed primers sets, and SYBR Green master mix (Applied Biosystems). The gene expression was evaluated as 2^−ΔΔCT^ and plotted in the graph using GraphPad Prism software.

### RNA extraction/sample preparation and bulk-RNA sequencing and bioinformatics

Ovarian cancer cells (CP70 CD55 OE and KO) were grown on 100 mm Petri dishes to 70% confluence. Cells were washed two times with D-PBS and cells and harvested. Total RNA was extracted using RNeasy kit (Qiagen Inc). RNA concentration and RNA integrity number (RIN ≥ 7) were measured using Bioanalyzer. High-quality RNA was processed for library preparation and sequencing using methods published by Sangwan [19]. Raw sequencing reads were quality-trimmed using trimmomatic pipeline [20]. Quality filtered reads were mapped to the reference genome (GRCm39) [21] using STAR aligner [22], and gene expression levels were quantified using the count module in RNA-Seq by Expectation-Maximization (RSEM) v.1.3.3. Raw gene count matrices were prefiltered and processed for downstream analysis using methods published by Sangwan [19, 23]. Briefly, differential gene expression and pathway enrichment analysis were performed using edge [24] and topGO package [25]. Gene set enrichment analysis was performed using the command line pre-ranked GSEA application downloaded from the Broad Institute’s website with Mouse MSigDB (v2022.1.Mm) as a reference database [26].

### In vivo experiments in NOD-*scid* IL2Rgamma^**null**^(NSG) mice

Pre-clinical studies were performed in NSG mice under a protocol reviewed and approved (Protocol number# 2986) by the Cleveland Clinic Institutional Animal Care and Use Committee (IACUC). Briefly, CP70 CD55OE, KO, ∆1234 and ∆ST cells were transduced with pCDH-EF1a-eFFly-mCherry lentivirus. FFly luciferase labeled (0.20 × 10^6^ cells/mice) cells were injected in NSG mice intraperitoneally. After 10 days mice were divided into eight cohorts. **(1)** OE Vehicle (*N* = 9), **(2)** OE Cisplatin (*N* = 9), **(3)** KO Vehicle (*N* = 9), **(4)** KO Cisplatin (*N* = 9), **(5)** ∆1234 Vehicle (*N* = 9), **(6)** ∆1234 Cisplatin (*N* = 9), **(7)** ∆S/T Vehicle (*N* = 9), **(8)** ∆S/T Cisplatin (*N* = 9). The dose of Cisplatin was 2 mg/kg twice a week. During the study, mice were kept in an isoflurane inhalation chamber to induce anesthesia. D-luciferin solution was injected and bioluminescence images of the tumor in each mouse were captured by IVIS Lumina (PerkinElmer). IVIS images were analyzed by Living Image Software (Caliper Life Sciences). The fold change in tumor growth was measured and plotted in the graph. At necropsy, tumors were harvested and fixed in paraformaldehyde solution for immunohistochemical analysis according to the earlier reported method [27]. Additionally, TUNEL assay was performed to visualize the extent of DNA fragmentation in mice tumor specimens according to the manufacturer protocol.

### Human ovarian tumor specimen collection and immunohistochemical analysis

Human ovarian tumor samples (FFPE tissue sections) were collected from patients in the Cleveland Clinic Foundation (IRB 19–185) and immunohistochemistry of the FFPE tissue sections was performed. Briefly, tissue slides were put into Histo-Clear (National Diagnostics) to remove the paraffin. Tissue sections were rehydrated in graded ethyl alcohol (100%, 95%, 80%, and 60% ethanol). Antigen retrieval was performed by boiling the tissue sections in Tris–EDTA buffer (pH9). After cooling, sections were incubated in 0.01% triton X-100 for 10 min for permeabilization. Tissue sections were then blocked in 5% goat serum for one hour and primary antibodies (CD55 1:500 dilution, Proteintech, and EMD Millipore) were added to the sections and incubated overnight at 4ºC in a humidified chamber. Following antibody incubation, tissue sections were washed three times with PBS. Peroxidase labeled polymer antibody (Vector Labs) was added to the tissue sections and incubated for 30 min. After washing, DAB chromogen was added for 5 min. Slides were washed with PBS three times and sections were then counterstained with hematoxylin to visualize the nuclei.

### Software and statistical analysis

Image J software and Graph pad prism were utilized for image processing and graphical representation. Every experiment was performed at least three times. For multiple group analyses, One-way Analysis of Variance (ANOVA) was performed with Tukey’s post hoc comparison to determine p values (* *p* < 0.05, ** *p* < 0.01, *** *p* < 0.00, **** *p* < 0.0001). In other indicated assays, unpaired t test (* *p* < 0.05) was performed to determine the statistical significance. ELDA software was used to measure the stem cell frequency of ovarian cancer cells.

## Results

### CD55 is localized in the nucleus of ovarian tumors

**Fig. 1:**
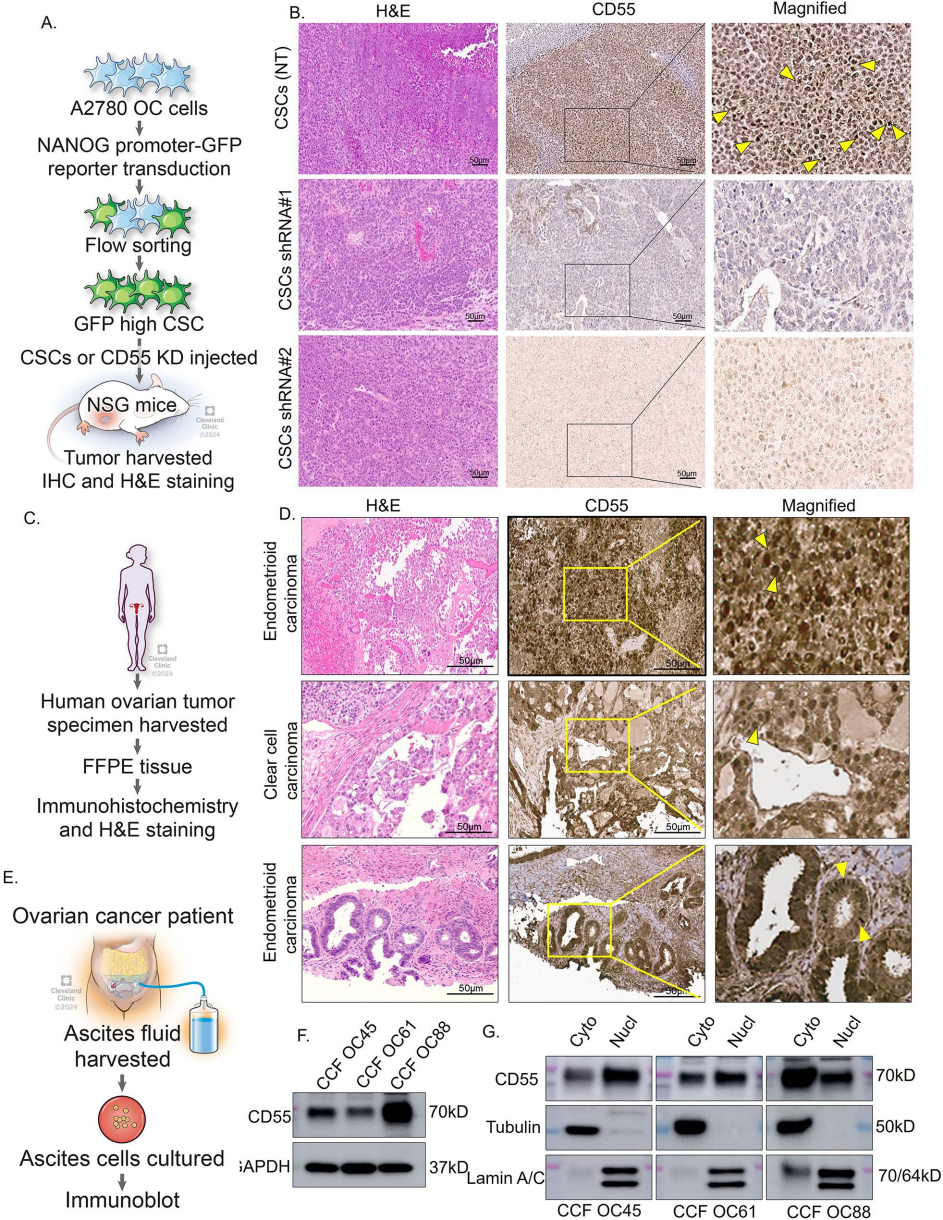
CD55 protein localizes in the nucleus of human ovarian cancer cells. (**A**) Generation of A2780 cancer stem cells from A2780 ovarian cancer cells using an established NANOG-GFP reporter system. Cancer stem cells were injected into NSG mice and tumors harvested at necropsy and fixed for FFPE analysis. (**B**) Tumor specimen from A2780 CSCs non-targeted control (NT), shRNA#1 and shRNA#2 were processed for hematoxylin & eosin (H&E) and immunohistochemical analysis for CD55. Yellow arrowheads denote CD55 nuclear staining. (**C**) Human ovarian tumor specimen (Clear cell carcinomas and Endometrioid ovarian carcinoma) collected and fixed. (**D**) Hematoxylin and eosin staining, and CD55 immunohistochemistry were performed. Yellow arrowheads denote CD55 nuclear staining. (**E, F**) CD55 protein expression in ascites cells from chemoresistant OC patients. CCF OC45, CCF OC61 and CCF OC88 ascites cells cultured, harvested, and lysed for immunoblot analysis of CD55. (**G**) CCF OC45, CCF OC61 and CCF OC88 cells were harvested, fractionated for nuclear and cytoplasmic pools, and immunobloted for CD55.  Lamin A/C and tubulin used as nuclear and cytoplasmic loading controls, respectively

Multiple studies, including ours, link CD55 to the maintenance and propagation of cancer stem cells (CSCs) and to increased chemoresistance and tumor recurrence [3, 28–31]. Our studies were the first to show induction of self-renewal and therapeutic resistance in ovarian endometrioid cancer (OEC) that supported findings that blocking LCK sensitizes OEC to cisplatin chemotherapy [3, 32, 33]. We sought to test whether CD55 can be informative for assessing ovarian cancer outcomes in primary patient formaldehyde fixed paraffin embedded (FFPE) specimens including localization to the CSC niche using Immunohistochemistry (IHC) analysis. We initiated these studies by performing IHC analysis of specimens available from our previous studies [3]. Xenograft tumors from NSG mice injected with A2780 OEC cells enriched for CSCs were stained with CD55 antibody (Fig. 1A). We reasoned that using CSC tumors known to express elevated levels of CD55 would provide an enriched specimen for developing our antibody staining protocols [3]. Unexpectedly, CD55 was detected in the nucleus in a patchy distribution throughout the specimen (Fig. 1B). We tested the IHC staining using another CD55 antibody (EMD Millipore) and confirmed the nuclear localization of CD55 (Supplementary Fig. 1A). As specificity controls, we also stained sections from CD55 silenced tumors (CD55 shRNA#1 and CD55 shRNA#2, Fig. 1B). In contrast to CSC enriched A2780 tumors, little CD55 staining was detected in the cytoplasm and nucleus (Fig. 1B). Given the unexpected observation of nuclear CD55, we analyzed CD55 localization in patient specimens (Supplementary Table 2). We detected CD55 in the nucleus of endometrioid and clear cell ovarian carcinoma specimens albeit in a patchy nonuniform distribution of a subset of tumors suggestive of CSC niche expression (Fig. 1C, and D). Cytoplasmic/membranous CD55 was found uniformly in the specimens. We procured three specimens from patients that had undergone treatment at the Cleveland Clinic that were determined to be platinum-resistant (CCF OC45, CCF OC61, CCF OC88 Fig. 1E). The morphology of ascites cells from CCF OC45 and CCF OC61 appeared mesenchymal whereas CCF OC88 appeared epithelial (Supplementary Fig. 1B). All three specimens expressed CD55 in immunoblots of whole cell lysates (Fig. 1F), as well as in the cytoplasmic and nuclear fractions (Fig. 1G). Collectively, these findings indicate CD55 is present in the nucleus of a subset of ovarian cancer patient specimens including ascites from recurrent and resistant disease.

### CD55 localizes to the nucleus of ovarian cancer cell lines

**Fig. 2:**
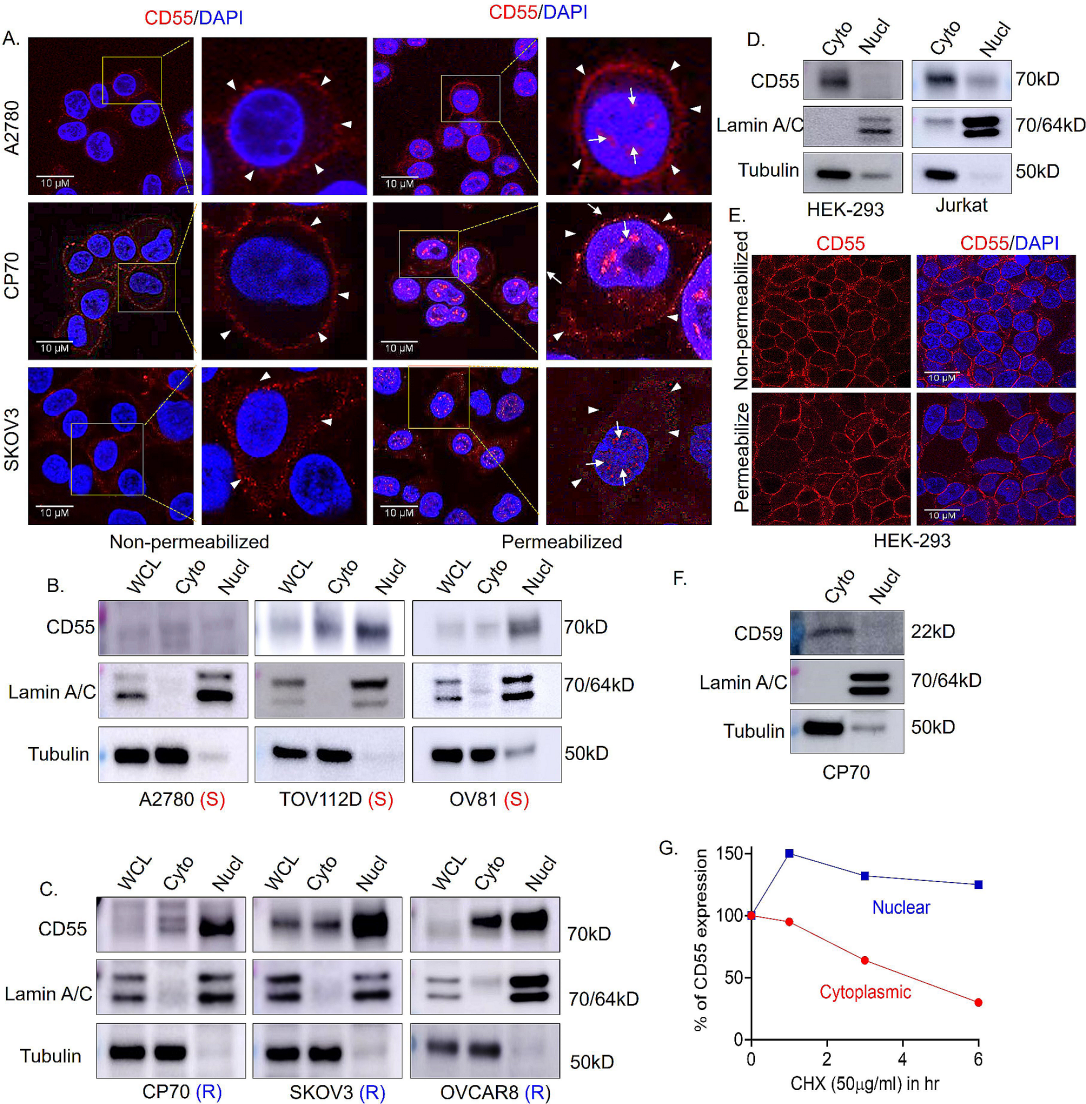
Cell surface CD55 is enriched in the nucleus of chemoresistant ovarian cancer cells. (**A**) A2780, CP70, and SKOV3 ovarian cancer (OC) were analyzed by immunofluorescence (IF) for CD55 protein localization. After fixation, cells were treated either with or without a permeabilizing agent (Triton X-100), followed by IF processing using a CD55 antibody. The cells were then counterstained with DAPI to visualize the nucleus. Cell surface CD55 expression is indicated with arrowhead and nuclear CD55 with an arrow. (**B, C**) Platinum sensitive (Designated as S) and resistant (Designated as R) ovarian cancer cells were lysed and whole cell, cytoplasmic, and nuclear fractions isolated followed by SDS-PAGE and immunoblotting for CD55 protein. Lamin A/C and Tubulin were used as nuclear and cytoplasmic marker respectively. (**D**) HEK293 and Jurkat cells were fractionated to enrich cytoplasmic and nuclear compartments, resolved by SDS-PAGE, and blotted for CD55. Lamin A/C was used as nuclear marker and Tubulin was used as cytoplasmic marker. (**E**) HEK293 cells were grown on coverslips, fixed with paraformaldehyde, treated with/without permeabilizing agent (Triton X-100) followed by IF for CD55. DAPI counterstaining was used to detect the nuclei. (**F**) Cytoplasmic and nuclear expression of CD59, a GPI-anchored protein in CP70 cells. (**G**) CP70 cells were treated with cycloheximide (50 µg/ml) for 0, 1, 3, and 6 h, followed by cell fractionation for cytoplasmic and nuclear isolation. Samples separated on SDS-PAGE followed by western blotting for CD55 protein expression corrected to 0 time point. Percentage of CD55 expression relative to initial CD55 protein are presented (Blots shown in supplementary Fig. 1I). Tubulin and lamin A/C immunoblots show relative enrichment of cytoplasmic and nuclear fractions respectively

The serendipitous discovery of nuclear CD55 (nCD55) in tumors led us to re-analyze the localization of CD55 in ovarian cancer cell lines with a focus on the nucleus. CD55 is a GPI-anchored protein localized to cell-surface lipid rafts or microdomains [3]. In absence of cell-permeabilizing agent (0.01% triton-X-100), CD55 was clearly visible at the plasma membrane in A2780, CP70, and SKOV3 ovarian cancer cells by immunofluorescence (IF) (Fig. 2A). However, upon cell-permeabilization, CD55 demonstrated a punctate localization in the nucleus, in addition to its presence at the plasma membrane (Fig. 2A). To further investigate the nuclear localization, cellular fractionation followed by immunoblotting for CD55 was performed. A2780 and TOV112D are human ovarian cancer cell lines derived from human ovarian cancers and OV81 was developed from a patient-derived xenograft. A2780, TOV112D, and OV81 are platinum-sensitive cells [3, 18, 34, 35], whereas CP70, SKOV3, and OVCAR8 are platinum-resistant cells [3, 34, 36]. Biochemical subcellular fraction followed by immunoblotting revealed strong nuclear enrichment of CD55 protein relative to the cytoplasm in platinum-resistant cancer cells CP70, SKOV3, and OVCAR8 compared to the platinum-sensitive cells (Fig. 2B and C, Supplementary Fig. 2A). The findings indicate nuclear CD55 is associated with chemoresistance as the expression of the protein is increased in cisplatin-resistant (CP70 and SKOV3) compared to naïve (A2780 and TOV112D) ovarian cancer cells (Supplemental Fig. 2B, E). Resistance and sensitivity to cisplatin in CP70, SKOV3, OVCAR8, A2780, OV81, and TOV112D were validated in dose-response studies (Supplemental Fig. 2C). We next overexpressed CD55 in parental CP70 and SKOV3 and found increased CD55 at the cell surface and nucleus by immunofluorescence analysis (Supplemental Fig. 2D) and immunoblots confirmed endogenous expression as well as overexpression of CD55 in the nucleus (Supplemental Fig. 2F). To further confirm our findings, we generated CD55 knock out (KO) CP70 cells using CRISPR/CAS9. In KO cells, there was no expression of CD55 protein found in cytoplasm or the nucleus providing evidence that immunoblot and immunofluorescence expression is specific (Supplemental Fig. 2G). As all studies were performed with Proteintech generated antibodies, we validated the findings with EMD Millipore antibody and confirmed the localization of CD55 in the nucleus (Supplemental Fig. 2H). To date, CD55 has been found to be exclusively localized to the cell surface. Jurkat and HEK293 cells were used as negative controls for nuclear localization of CD55 as these cells are reported to express CD55 solely at the cell surface [37–39]. As such, we analyzed cytoplasmic and nuclear expression of CD55 in HEK293 and Jurkat cells by immunoblotting and IF, and found prominent cell surface CD55 localization, while nuclear localization was low to undetectable (Fig. 2D and E, Supplementary Fig. 2I). To further investigate the specificity of CD55 nuclear localization, we investigated whether CD59, a CD55 related GPI-anchored membrane complement regulatory protein, could also localize to the nucleus [40]. Unlike CD55, CD59 is exclusively found in the cytoplasmic fraction (Fig. 2F, Supplemental Fig. 3A). Collectively, these findings support the hypothesis that CD55 nuclear localization is unique to cisplatin-resistant OC cells.

Given the unexpected localization, we hypothesized that the cytoplasmic and nuclear CD55 pools would have differential stability. We assessed the stability of CD55 protein after treatment of the cell with the protein translation inhibitor cycloheximide for 1, 3, and 6 h followed by fractionation to nuclear and cytoplasmic pools and immunoblotting for CD55. We determined that cytoplasmic CD55 was less stable, with a protein half-life of ~ 4.2 h, while nCD55 protein was stable (Fig. 2G, Supplemental Fig. 3B). The differential stability may account for a higher steady state of CD55 protein in the nucleus.

### Nuclear CD55 is glycosylated and derived from the cell surface pool of CD55

**Fig. 3:**
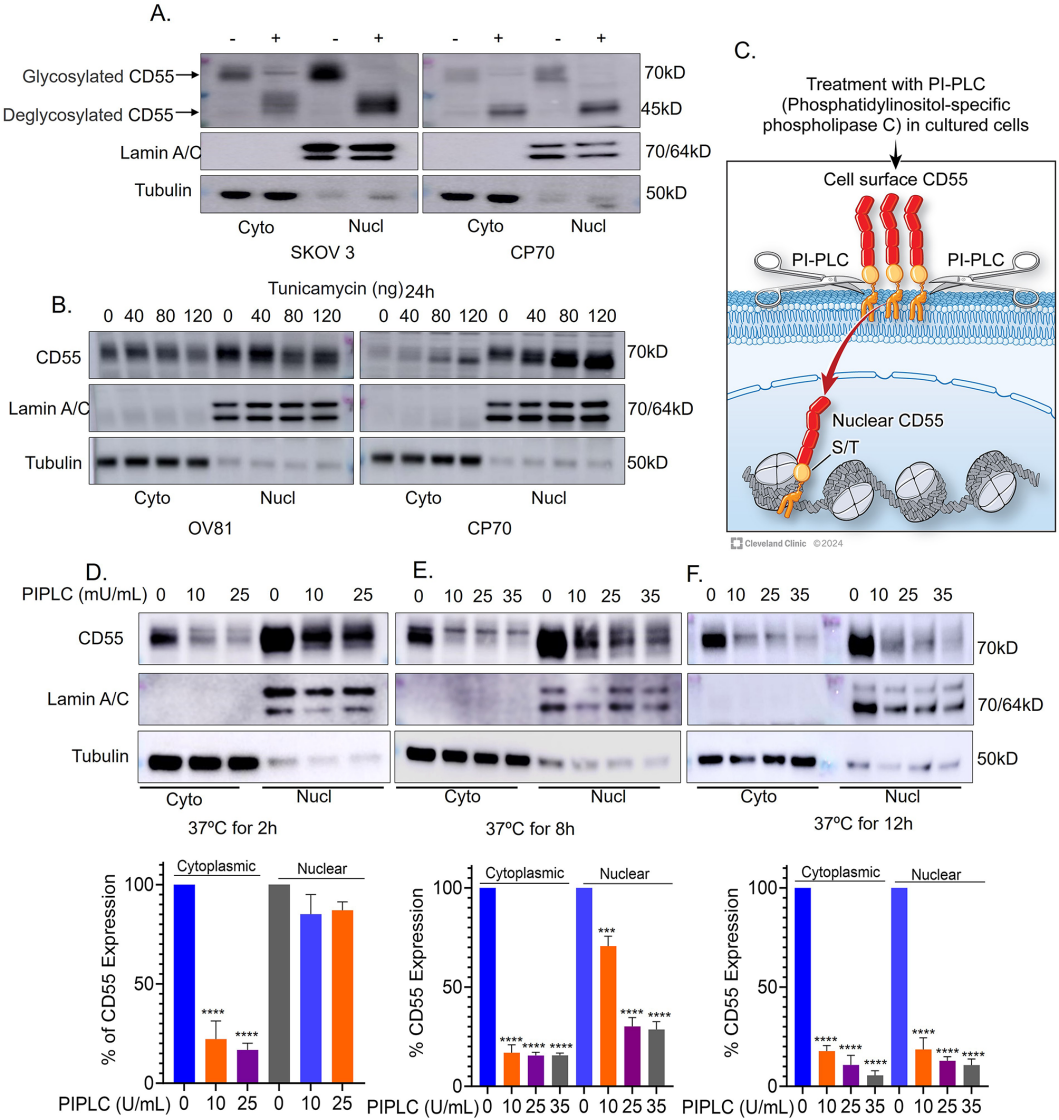
Nuclear CD55 is glycosylated and originates from the cell surface. (**A**) Cytoplasmic and nuclear proteins from ovarian cancer cells were fractionated. Fractionated proteins were then treated with protein deglycosylation mix II enzyme to remove glycosylation from CD55 proteins. Protein samples were processed for immunoblot analysis. (**B**) Ovarian cancer cells (OV81 and CP70) were subjected to 24-hour tunicamycin treatment. Subsequently, cytoplasmic, and nuclear proteins were fractionated, and CD55 protein expression was assessed through immunoblot analysis. (**C**) PIPLC treatment strategy to shed surface CD55 protein. (**D, E**, and **F**) CP70 cells were treated with increasing dose of PIPLC (0, 10, 25, 35 Unit/ml) at 37 °C for 2, 8, and 12 h. At indicated times, cells were harvested and fractionated, followed by separation by SDS-PAGE and CD55 immunoblotting. The resulting blots were quantified using Image J software. Data are representative of an experiment that was repeated 3 times

We assessed whether nuclear CD55 is structurally glycosylated similar to surface CD55. Cell surface CD55 has been previously shown to be N-glycosylated and heavily O-glycosylated [41]. Protein glycosylation is a common post-translational modification that can direct protein localization. We assessed the glycosylation status using a protein deglycosylation enzyme kit (NEB, USA) to cleave O-glycosylation polysaccharides from CD55. Whole cell lysate, cytoplasmic, and nuclear fraction were treated and analyzed by SDS-PAGE and immunoblotted for CD55 (Fig. 3A and Supplemental Fig. 4A). Both cytoplasmic and nuclear CD55 proteins exhibited increased migration to an equivalent extent indicating they are both heavily O-glycosylated. As CD55 is N-glycosylated, we treated cells without or with increasing concentrations of tunicamycin (N-glycosylation inhibitor), harvested cells, and separated into cytoplasmic and nuclear fractions, followed by SDS-PAGE and immunoblotting for CD55. Similar to O-glycosylation, cytoplasmic and nuclear CD55 exhibited the same increase in migration indicating that both pools are structurally similar (Fig. 3B). Our findings are corroborated with previous findings that glycosylated form of proteins are present in the nucleus [42].

Finding CD55 in the nucleus led us to assess the trafficking of cytoplasmic and nuclear CD55 pools. We first analyzed the origin of nuclear CD55, investigating whether it traffics from the cell surface and then to the nucleus. To address this, we treated CP70 cells with PIPLC at 37 °C for varying durations of 2, 8, and 12 h. PIPLC cleaves GPI-anchored proteins from the cell surface [43]. We treated CP70 cells with PIPLC and found complete depletion of the cytoplasmic pool after 2 h, accompanied by minimal reduction in the nuclear pool (Fig. 3C, D). Notably, the 8 and 12-hour treatment groups exhibited substantial depletion in both the cytoplasmic and nuclear CD55 pools (Fig. 3E and F). Similar results were observed in other ovarian cancer cells OV81 (Supplemental Fig. 4B, C). Although the turnover of CD55 in OV81 cells is more rapid than in CP70. This in turn results in a more rapid decrease of cell surface and nuclear CD55 pools with a delay in the reduction of nuclear CD55 compared to the cell surface pool. These observations collectively support the concept that nuclear CD55 likely originates from a cytoplasmic pool.

### Serine/Threonine (S/T) rich domain is sufficient for nuclear localization of CD55

**Fig. 4:**
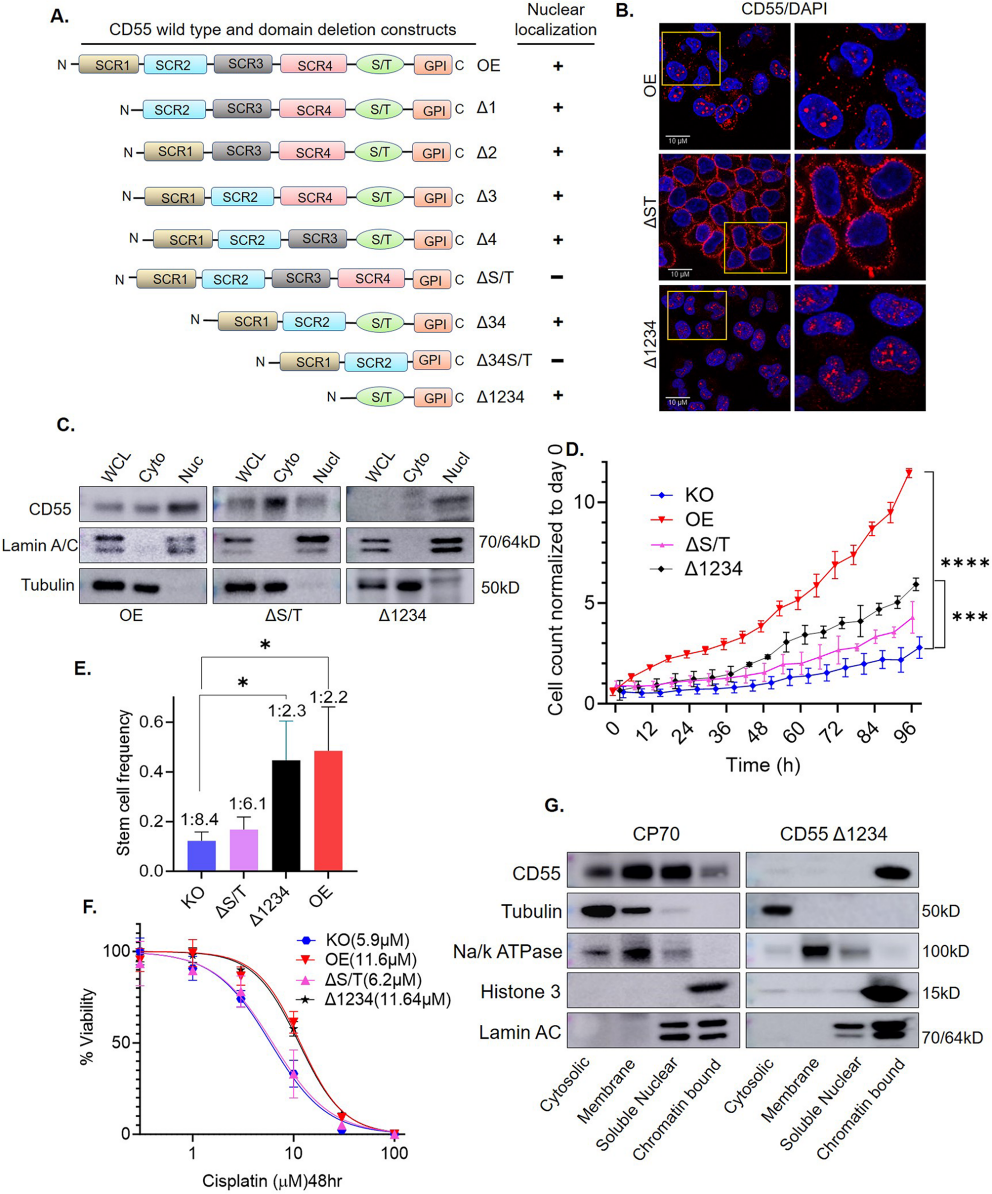
CD55 S/T domain binds chromatin and drives cell proliferation, CSC frequency, and cisplatin resistance. (**A**) Wild type CD55 and domain deletion mutants were engineered and cloned into lentiviral vector, pLenti CMV Puro DEST. Lentiviruses were transduced into CD55 CRISPR KO CP70 cells and stable cell lines generated. Cells were cultured and fractionated to obtain cytoplasmic and nuclear pools. Cytoplasmic and nuclear fractions were separated on SDS-PAGE and immunoblotted for CD55 (Blots shown in supplementary Fig. 5A). Summary of findings displayed as either positive (+) or negative (-) for nuclear localization. (**B**) Immunofluorescence analysis of CD55 OE, ΔST, and Δ1234 (S/T only) transduced CP70 cells. CD55 protein localization was shown in red color. Nucleus was counter stained with DAPI (Blue). (**C**) CD55 immunoblot of CD55 OE, ΔST, and Δ1234 transduced CP70 KO cells. LaminA/C was used as loading control for nuclear fraction and Tubulin was used as a control for cytoplasmic fraction. (**D**) Cell proliferation analysis using Incucyte. KO, OE, and mutants ΔST, Δ1234 were analyzed over a 4-day period. (**E**) Stem cell frequency in tumorspheres was analyzed by limiting dilution assay. One way ANOVA was performed, and Tukey’s multiple comparison test was performed to determine p values (* *p* < 0.05, ** *p* < 0.01, *** *p* < 0.001, **** *p* < 0.0001). (**F**) Cisplatin sensitivity assay of OE, KO, and mutants ΔST, Δ1234 transduced CP70 cells. Data was analyzed with GraphPad Prism and IC_50_ values are indicated in parentheses. (**G**) CP70 parental and ∆1234 cells were harvested and cytosolic, membrane, soluble nuclear, and chromatin bound protein fractions were analyzed by immunoblot for CD55. Tubulin (Cytosolic), Na^+^/K^+^ ATPase (Membrane), Histone 3 (Chromatin) and Lamin AC (Nuclear) were used as loading markers for each fraction

Our study showed that CD55 traffics to the nucleus. To identify the domains necessary for nuclear trafficking, we generated CD55 domain mutants with deletions of individual SCR and S/T domains as well as a few combinations (Fig. 4A). The GPI-domain is necessary for cell-surface targeting of CD55, and the GPI anchor code was present in all CD55 mutants generated. Full length CD55 (OE) and the deletion mutants were transduced into CD55 KO CP70 cells generated by CRISPR/Cas9. We screened nuclear localization by immunoblots as well as by IF of transduced cells. The analysis indicated that mutants lacking the S/T domain were restricted to the cytoplasm and cell surface, whereas mutants that contained S/T trafficked to the nucleus (Summarized in Fig. 4A). CD55-OE was localized to the cell surface and nucleus, ΔS/T was localized in the cytoplasm relative to the nucleus, whereas Δ1234 (expressing only S/T domain and GPI anchored domain) is localized in the nucleus relative to the cytoplasm (Fig. 4B and C). Deletion mutants Δ1, Δ2, Δ3, Δ4 and Δ34 containing the S/T domain localized, as expected, in both the cytoplasm and nucleus (Supplemental Fig. 5A).

### Nuclear localization is necessary for CD55 induction of self-renewal and platinum-resistance

CD55 mutants that are enriched in the nucleus or cytoplasm allowed us to probe for oncogenic roles related to differential localization. We assayed cell proliferation rates using the IncuCyte® cell count proliferation assay system and determined that transduction of OE in CD55 CRISPR KO CP70 cells led to a four-fold increase in proliferation (Fig. 4D), as expected. ΔS/T-transduced in KO cells exhibited a small increase in proliferation rate that was lower than OE, while Δ1234 (includes the S/T domain) exhibited an intermediate proliferation rate (Fig. 4D**)**. Similarly, the proliferation rates of Δ1, Δ2, Δ3, Δ4, Δ34 and Δ34S/T exhibited similar proliferation rates as OE when transduced into KO cells (Supplemental Fig. 5B). As CD55 induces CSCs in ovarian cancer cells [3], we assessed the impact of nuclear enrichment on CSC frequency using our established spheroid assays [2]. Overexpression of CD55 in the CD55 CRISPR KO cells led to a four-fold increase in CSC frequency (Fig. 4E). ΔST did not increase CSC frequency, while Δ1234 exhibited a similar increase in CSC frequency as OE (Fig. 4E, Supplementary Fig. 5D). Representative images of spheroids from CD55 OE, KO, ∆1234 and ∆S/T presented in Supplemental Fig. 5D. Likewise, the CSC frequency of Δ1, Δ2, Δ3, Δ4, and Δ34 mutants resembled the frequency observed in CD55 OE transduced cells (Supplemental Fig. 5C). As CD55 can induce chemoresistance in ovarian cancer cells, we next performed cisplatin sensitivity assays with the mutants introduced in the CD55 KO background. Overexpression of CD55 led to a two-fold increase in chemoresistance (Fig. 4F). ΔS/T and Δ34S/T did not increase chemoresistance, while Δ1234 (Fig. 4F), Δ1, Δ2, Δ3, Δ4, and Δ34 exhibited similar increases in chemoresistance as OE (Supplemental Fig. 5E). Given the difference in stability of the cytoplasmic and nuclear pools, we analyzed stability of the individual mutants using cycloheximide treatment (Supplemental Fig. 5F, G, H). Our findings indicate that the S/T domain promotes the stability of CD55 protein in OC cells.

Our data demonstrate that specifically the S/T domain is sufficient for the nuclear localization of CD55 and induces CSCs and chemoresistance to the levels of CD55 OE. We next sub-fractionated the cytosolic fraction to cytosolic and membrane pools, and the nuclear fraction to soluble and chromatin sub-fractions. In parental CP70 cells, CD55 localized in all four sub-fractions including soluble nuclear, and chromatin-bound fractions. In CD55 KO cells transduced with a CD55 mutant containing only S/T and GPI domains (Δ1234), the protein was solely localized to the chromatin-bound fraction (Fig. 4G). The findings implicate the S/T domain of nuclear CD55 in chromatin-binding or modification and uncover a previously unknown chromatin-directed role of CD55.

### Nuclear CD55 induces chemoresistance in vivo

**Fig. 5:**
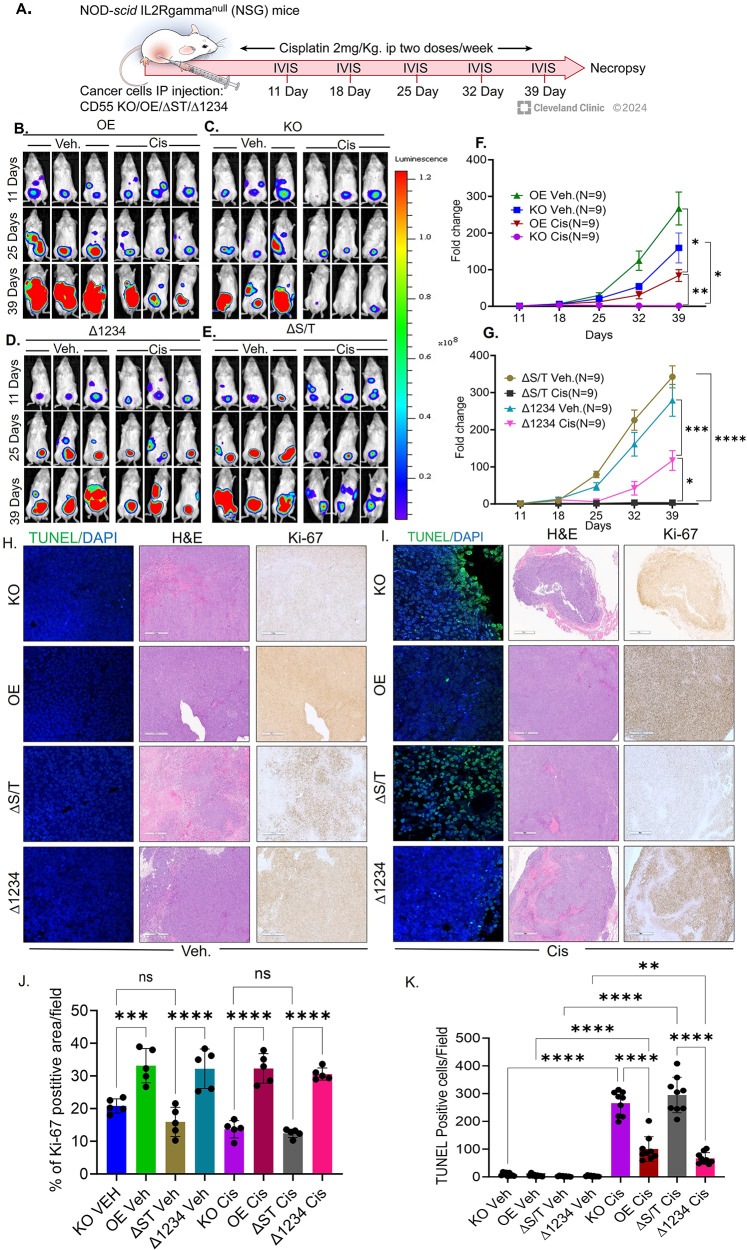
CD55 nuclear localization accelerates tumor growth and induce chemoresistance. (**A**) CP70 CD55 KO, OE, ∆1234, ∆S/T cells were intraperitoneally injected into NSG mice. Once tumors were detected, mice were randomized into either saline (Veh) or cisplatin (2 mg/kg) twice weekly. (**B**, **C**, **D**, **E**) Representative bioluminescence images of 3 tumor bearing mice from each cohort at 11, 25, and 39 days of the treatment in (B) OE cohort (C), KO cohort (D), ∆1234 cohort and (E) ∆S/T cohort. (**F**) Tumor growth kinetics of KO vs. OE mice treated with cisplatin or veh. (**G**) Tumor growth kinetics of ∆1234 vs. ∆ST mice treated with cisplatin or veh. (*N* = 9 mice/treatment). (**H**) Tumor FFPE sections from Veh treated KO, OE, ∆S/T and ∆1234 mice were analyzed for apoptosis (TUNEL assay), cell proliferation (Ki-67 immunohistochemistry, and H&E staining. (**I**) Tumor FFPE sections from Cisplatin treated KO, OE, ∆S/T and ∆1234 mice were analyzed for apoptosis (TUNEL assay), cell proliferation (Ki-67 immunohistochemistry, and H&E staining. (**J**) Analysis of Ki-67 positive cells in FFPE sections from KO, OE, ∆S/T and ∆1234 mice. Representative data from 5 different fields of Ki-67 FFPE section of each treatment group. (**K**) Analysis of TUNEL positive cells in FFPE sections from KO, OE, ∆S/T and ∆1234 mice. Representative data from 10 different fields of Ki-67 FFPE section of each treatment group. One way ANOVA was performed, and Tukey’s multiple comparison test was performed to determine p values (* *p* < 0.05, ** *p* < 0.01, *** *p* < 0.001, **** *p* < 0.0001)

We analyzed ∆1234 (nuclear restricted) and ∆ST (nuclear excluded) mutants to ascertain the physiological relevance of CD55 nuclear localization in tumor growth and chemoresistance in vivo. CD55 OE, KO, ∆1234 and ∆S/T mutant containing cancer cells were injected into the mice. Once tumors were detected, mice were injected with saline or cisplatin (Fig. 5A). IVIS imaging was captured to visualize the tumor growth of the individual mice (Fig. 5B, C, D, E, F and G Supplemental Figs. 6, 7). As control, we compared the tumor growth and chemosensitivity to OE and KO cells. OE exhibited rapid tumor growth compared to KO cells (Fig. 5B, C and F). IVIS images were quantified followed by growth kinetics analysis. The data indicate OE tumor growth is accelerated compared to KO though not significantly whereas cisplatin completely suppresses tumor growth in KO compared to OE (Fig. 5F). IVIS images were quantified and indicate that while tumor growth in ∆1234 and ∆S/T are not significantly different, cisplatin is sufficient to suppress tumor growth in the ∆S/T mutant cells (Fig. 5D, E, G). We further performed immunohistochemical analysis of Ki-67, an established tumor proliferation marker in mice tumor sections. Interestingly, we found that vehicle treated CD55 OE and∆1234 tumors expressed significantly higher expression of Ki-67 compared to CD55 KO and ∆S/T tumors (Fig. 5H and J). More importantly, in Cisplatin treated cohorts, CD55 OE and, ∆1234 displayed high Ki-67 expression compared to CD55 KO and ∆S/T tumors (Fig. 5I and J). Next, we assessed the extent of apoptotic DNA fragmentation induced by Cisplatin using TUNEL assays. All vehicle treated cohorts showed no TUNEL positivity. Interestingly, Cisplatin treated CD55 OE and, ∆1234 tumors showed significantly lower TUNEL positivity as compared to Cisplatin treated CD55 KO and ∆S/T tumors (Fig. 5H, I and K). Collectively, these data support the hypothesis that nuclear localization of CD55 is sufficient to promote chemoresistance in ovarian cancer in vitro and in vivo.

## Nuclear CD55 binds and attenuates the epigenetic reader and tumor suppressor ZMYND8

**Fig. 6:**
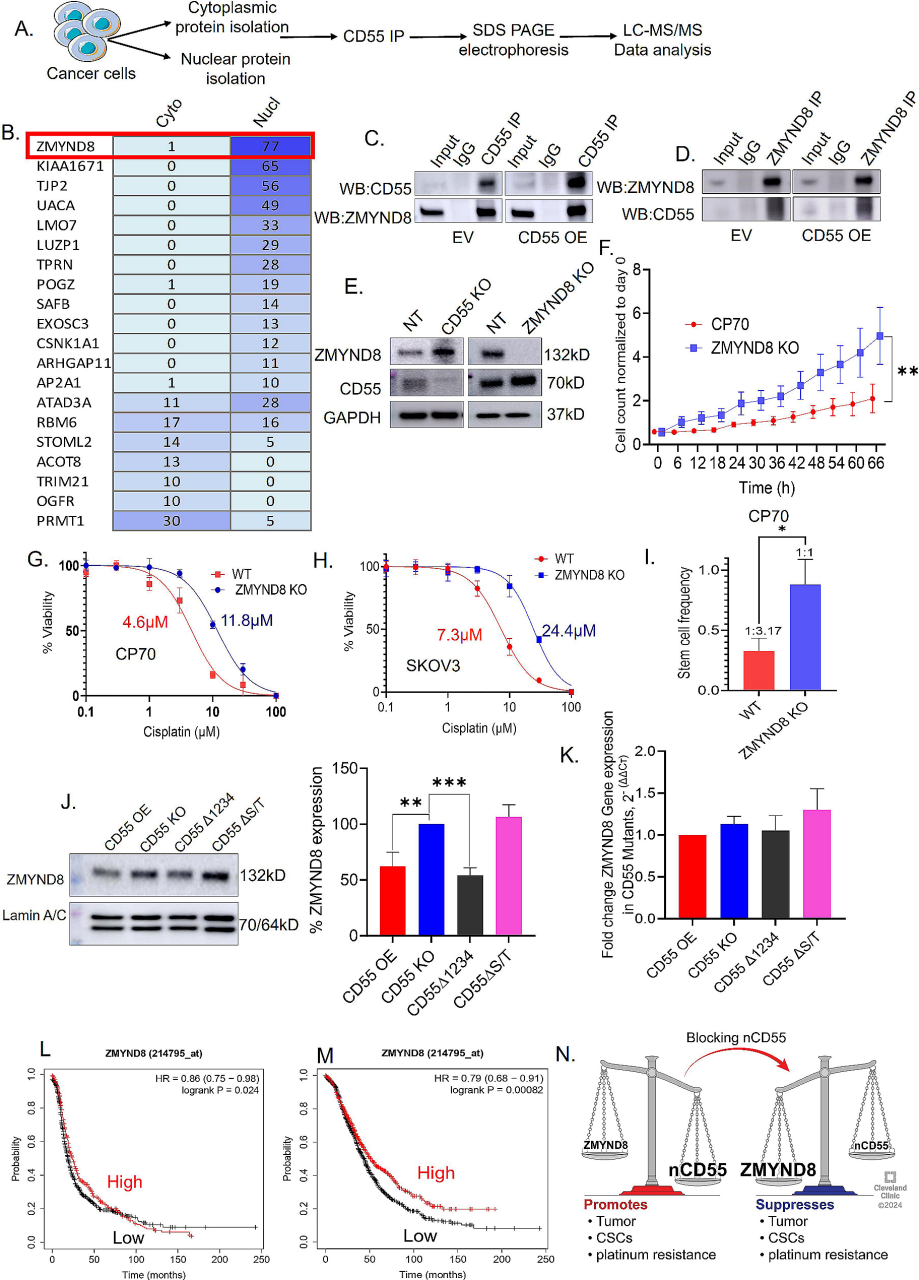
ZMYND8 is a binding partner of nuclear CD55. (**A**, **B**) CP70 cells were cultured and cytoplasmic and nuclear proteins were extracted. CD55 protein was immunoprecipitated (IP) from cytoplasmic and nuclear fractions. The immunoprecipitated protein samples were used to run SDS PAGE. The gels were sent for LCMS analysis to detect binding partners of CD55 protein. Spectral counts obtained from LCMS analysis indicated relative abundance of CD55 binding partners. ZMYND8 was identified as the most abundant binding partner of CD55 protein in the nucleus. (**C**) CP70 cells were transduced with empty vector (EV) or CD55, harvested and lysed followed by immunoprecipitation with CD55 and western blot for CD55 and ZMYND8. (**D**) CP70 cells, transduced with empty vector (EV) or CD55 were harvested and lysed followed by immunoprecipitation with ZMYND8 and western blot for ZMYND8 and CD55. (**E**) Expression of ZMYND8 and CD55 in KO cells. (**F**) ZMYND8 was knocked out by CRISPR, and cell proliferation analyzed by Incucyte. (**G**) Cisplatin sensitivity was assayed in CP70 cells. 95% Confidence Interval (CI) is 10.77–12.97. in ZMYND8 KO, 3.97–5.41 in parental cells. (**H**) Cisplatin sensitivity assay. (**I**) CSC frequency assay. Unpaired t test * *p* < 0.05. (**J**) CP70 cells (CD55 OE, CD55 KO, CD55∆1234, and CD55∆ST) were grown and immunoblot was performed. Quantification of ZMYND8 expression was also performed using image J software. One way ANOVA was performed, and Tukey’s multiple comparison test was performed to determine p values (* *p* < 0.05, ** *p* < 0.01, *** *p* < 0.001, **** *p* < 0.001). (**K**) Real time PCR was performed to check ZMYND8 gene expression in CP70 cells. (**L**) Progression free survival in relation to ZMYND8 expression in 1435 HGSOC and endometrioid patients determined using KMPlot. Number at risk, low = 1017 and high = 418. Hazards ratio and Logrank P indicated in the graph. (**M**) Overall survival in relation to ZMYND8 expression in 516 HGSOC and endometrioid patients determined using KMPlot. Number at risk, low = 1140 and high = 692. Hazards ratio and Logrank P indicated in the graph. (**N**) Reciprocal regulation of CD55 and ZMYND8

The presence of CD55 in the nucleus, the interaction of nCD55 with chromatin, and the requirement of nCD55 for the promotion of chemoresistance in OC cells and tumors led us to hypothesize that nCD55 interacts with a nuclear protein complex to promote stemness and chemoresistance. To identify nCD55 binding partners, we immunoprecipitated CD55 from cytoplasmic and nuclear fractions, validated CD55 and sequenced all co-precipitating proteins. CD55 was the primary protein in the 70 kDa region of the gel based on MS/MS sequencing (Supplemental Fig. 8A). One of the major CD55 binding partners identified in the nuclear fractions by mass spectral analysis was ZMYND8, also known as RACK7 or PKCBP [44–46] (Fig. 6A and B). The CD55-ZMYND8 interaction was validated by co-immunoprecipitation (co-IP) first with CD55 IP followed by immunoblot for ZMYND8 as well as IP for ZMYND8 and immunoblot for CD55. Results demonstrated that in both empty vector (EV) and CD55 OE transduced CP70 cells CD55 was sufficient to co-IP ZMYND8 (Fig. 6C). Likewise, ZMYND8 was capable of co-precipitating CD55 (Fig. 6D). We next assessed the impact of CRISPR mediated KO of CD55 on ZMYND8 and found that ZMYND8 expression increased (Fig. 6E). In contrast, CRISPR-mediated KO of ZMYND8 lead to increased expression of CD55 (Fig. 6E). As ZYMND8/CD55 reciprocally regulated their expression, we investigated the impact of ZMYND8 KO on cell proliferation and determined that suppression of ZYMND8 lead to increased cell proliferation (Fig. 6F) similar to what was observed when CD55 was overexpressed in CD55 KO CP70 cells. Likewise, ZMYND8KO led to increased chemoresistance in CP70 (Fig. 6G) and SKOV3 cells (Fig. 6H) and increased CSCs (Fig. 6I). The IC_50_ in SKOV3 increased from 7.3 µM to 24.4 µM with a similar increase in CP70 from 4.6 µM to 11.8 µM in NT compared to KO cells, respectively (Fig. 6G and H). Further we analyzed ZMYND8 protein expression in CP70 CD55 OE, CD55 KO, CD55∆1234, and CD55∆S/T cells. In CD55 KO cells, ZMYND8 expression is increased compared to CD55 OE group. Interestingly, CD55∆S/T group also showed elevated ZMYND8 expression whereas CD55∆1234 showed no increase in ZMYND8 protein (Fig. 6J). This finding suggests that nCD55 protein suppresses ZMYND8 protein expression. However, CD55 OE, KO, ∆1234, and ∆S/T did not alter ZMYND8 transcript levels, indicating CD55-mediated regulation of ZMYND8 is post-transcriptional (Fig. 6K). The increases in CSC and chemoresistance of ZMYND8 KO cells are supported by Kaplan Meier survival analysis in HGSOC and endometrioid patients. Using KMPlot software [47], we determined high expression of ZMYND8 is correlated with increased progression free (Fig. 6L) and overall survival (Fig. 6M). Collectively, these studies indicate that CD55 and ZMYND8 reciprocally regulate one another to modulate CSC maintenance and ovarian tumor cell chemoresistance (Fig. 6N).

### Nuclear CD55 associates with PolyComb Repressive Complex 2 (PRC2) and derepresses H3K27me3 epigenetic marks

**Fig. 7:**
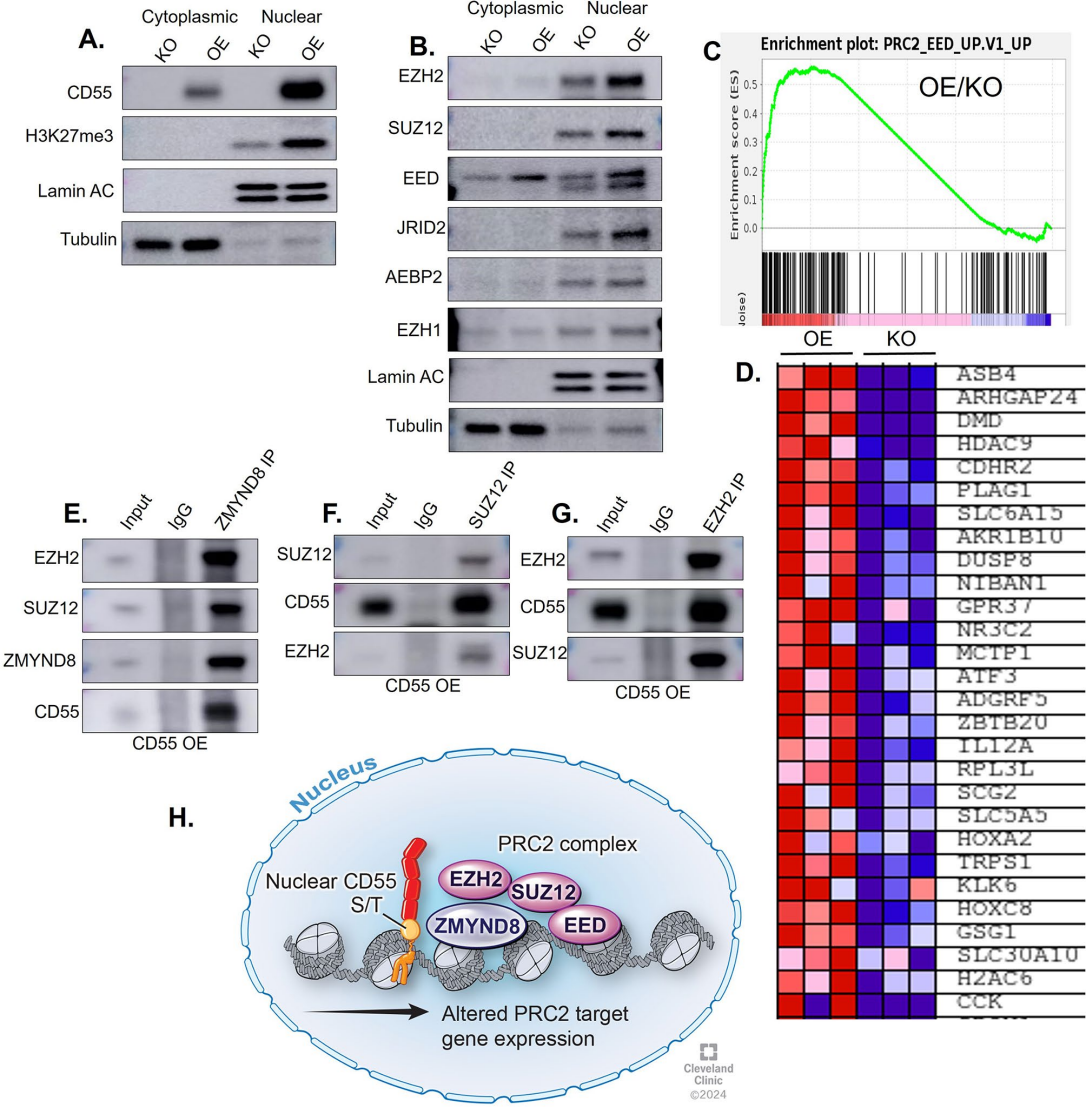
Nuclear CD55 associates with PRC2 members and regulates H3K27me3 mark. (**A**) Expression of H3K27Me3 in cytoplasmic and nuclear fractions of CP70 CD55 KO and CP70 CD55 OE cells. Lamin A/C was used as nuclear marker and tubulin was used as cytoplasmic loading control. (**B**) Expression of PRC2 complex members, EZH2, SUZ12, EED, JRID2, AEBP2, and EZH1 in cytoplasmic and nuclear fractions of CP70 CD55 KO and CP70 CD55 OE cells. (**C**) GSEA plot after bulk RNA sequencing in CP70 CD55 KO and OE cells. PRC2 target genes were positively correlated with CD55 overexpression. (**D**) Differential gene expression of PRC2 target genes in OE and KO cells. (**E**) CD55 OE cells were cultured, and nuclear fractions were prepared. ZMYND8 protein was immunoprecipitated followed by immunoblot analysis for EZH2, SUZ12, and ZMYND8. (**F**) Nuclear lysates of OE cells were prepared followed by SUZ12 protein immunoprecipitation and immunoblot analysis of SUZ12, CD55, and EZH2. (**G**) Nuclear fractions were prepared from OE cells followed by immunoprecipitation with EZH2 protein and immunoblot analysis of EZH2, CD55, and SUZ12. Each experiment was performed three times. (**H**) CD55 interacts with ZMYND8 and PRC2 complex to modulate PRC2 target genes in ovarian cancer

**Fig. 8:**
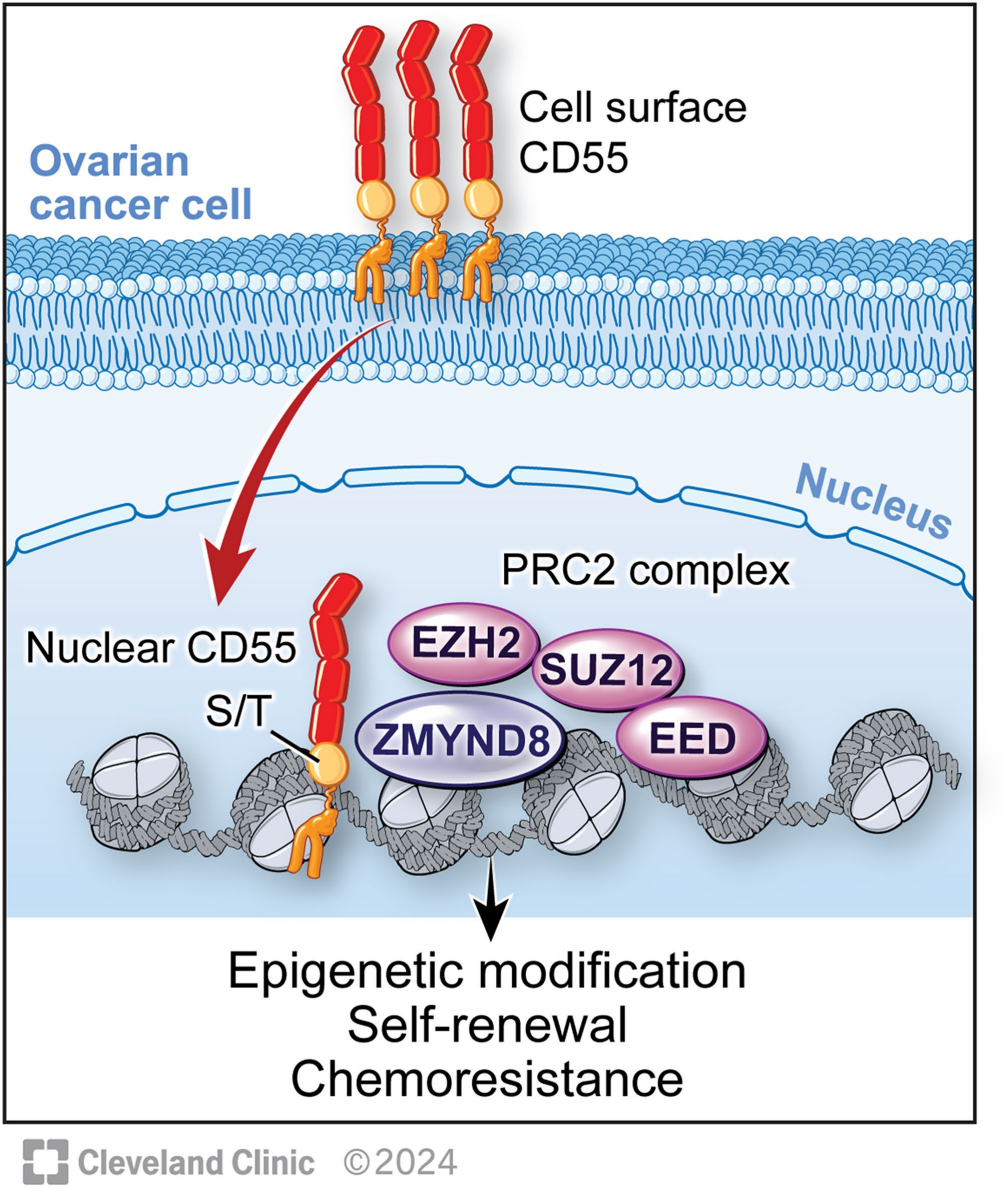
CD55 induces CSC activity and chemoresistance in Ovarian Cancers. Nuclear CD55 traffics to the nucleus from the cell surface. The S/T domain of CD55 is necessary for nuclear trafficking. In the nucleus, CD55 binds chromatin and interacts with ZMYND8 and PRC2 members and regulates PRC2 target gene expression. Nuclear CD55 drives epigenetic modifications, self-renewal, and platinum resistance in ovarian cancer cells

As ZMYND8 represses deposition of the histone mark histone-3 lysine-27 trimethylation (H3K27Me3), we assessed whether CD55 derepresses H3K27me3 deposition in OE cells [45]. Previous studies indicate ZMYND8 represses H3K27Me3 through its association with PolyComb Repressive Complex 2 (PRC2) members EZH2 and JARID1D, and that this interaction is necessary for stem cell maintenance and cell identity [48]. We examined H3K27Me3 levels in CD55 KO CP70 cells and CD55 overexpressing cells and found that CD55 increased H3K27me3 levels in nuclear fractions (Fig. 7A). ZMYND8 and CD55 exhibit opposing impact on H3K27Me3. As such, we investigated whether PRC2 proteins are induced by CD55 (EZH2, SUZ12, EED, JRID2, EZH1, and AEBP2). Overexpression of CD55 resulted in increased protein expression of EZH2, SUZ12, EED, JRID2, and AEBP2 in cells and nuclear fractions (Fig. 7B). The findings complement studies in a breast cancer model demonstrating that ZMYND8 negatively regulates PRC2 target genes [48]. Our data shows that CD55 positively regulates PRC2 target genes, and this was also confirmed by bulk RNA sequencing (Fig. 7C and D). Next, we investigated whether CD55 forms complexes with PRC2 members. We performed a co-immunoprecipitation followed by immunoblot analysis in OE cells and found ZMYND8 forms a complex with EZH2, SUZ12, and CD55 (Fig. 7E). These findings are consistent with a previous report of ZMYND8 and EZH2 interaction [49]. Next, we performed a co-IP followed by immunoblot using SUZ12 antibody and observed interactions with CD55 and EZH2 (Fig. 7F). Likewise, immunoprecipitation of EZH2 indicated interactions with CD55 and SUZ12 (Fig. 7G). Our findings showed that CD55 can form a nuclear complex with ZMYND8 and PRC2 members SUZ12 and EZH2 in platinum-resistant ovarian cancer cells (Fig. 7H). In summary, our findings indicate nuclear localization of CD55 may drive CSC and chemoresistance via interactions with ZMYND8 and PRC2 complex members (Fig. 8).

## Discussion

CD55 is classically thought of as a GPI-anchored membrane-associated complement regulatory protein. Most studies indicate a role of CD55 at the cell surface in innate immunity, bacterial and viral entry as well as cancer. Our previous studies indicate CD55 is sufficient to induce cancer stem cells and promote chemoresistance in the endometrioid subtype of ovarian cancer [3]. We now identify that these oncogenic functions stem from an unexpected localization of CD55 in the nucleus of cancer cells and ovarian cancer patients. Our studies indicate that the S/T domain contains a nuclear trafficking code. This domain also is required for O-glycosylation. Currently, we do not have evidence to suggest that O-glycosylation is necessary for trafficking to the nucleus and this question will be resolved in future studies. In the nucleus, CD55 associates with chromatin and interacts with ZMYND8, an epigenetic regulator and tumor suppressor. nCD55 inhibits ZMYND8 protein expression leading to increased stem cell properties and chemoresistance. Collectively, the findings reveal a novel localization and function for CD55 in the nucleus, specific to ovarian cancer cells, that may broaden chemotherapeutic avenues for ovarian cancer.

Our findings identify CD55 in the nucleus and demonstrate that nCD55 associates with chromatin. This is unique to CD55 as CD59, a related complement regulatory protein localized in lipid rafts is not found in the nucleus. Our studies add to the growing literature indicating that cell-surface proteins can traffic to and accumulate in the nucleus, impacting nuclear function. This includes Epidermal Growth Factor Receptor (EGFR), which can traffic to the nucleus leading to resistance to chemotherapy, radiotherapy, and anti-EGF targeted therapies in multiple cancers [50]. Additionally, our findings complement studies showing that Folate Receptor-α, a GPI-anchored protein, can translocate from the lipid microdomains at the cell surface to the nucleus where it acts as a transcription factor [51]. However, our discovery of nuclear CD55 is, to our knowledge, the first description of cell surface complement regulatory proteins localizing to the nucleus and interacting with chromatin.

We determined the S/T domain of CD55 is sufficient for trafficking to the nucleus. This domain does not contain a canonical nuclear localization signal [52]. This is noteworthy given the domain is heavily O-glycosylated with no defined function [5]. Protein glycosylation is a hallmark of membrane-targeting and secretory pathways, originating at the endoplasmic reticulum and extensively sculpted at the Golgi [53, 54]. The S/T domain is unique to CD55 as other GPI-anchored proteins including the GPI-anchored CD59 lack this domain. CD55 is known to traffic to the cell surface via the ER-Golgi pathway where it is post-translationally modified by the addition of a GPI-anchor [55]. Surprisingly, nuclear CD55 is glycosylated like cytoplasmic CD55, and our data show that the glycosylation domain is necessary for trafficking. Our PI-PLC findings suggest that the CD55 route to the nucleus is via retrograde transport from the cell surface. We find that treatment with PI-PLC leads to delayed loss of nCD55 compared to cytoplasmic CD55 and supports the hypothesis that nCD55 derives from the cell surface pool.

The discovery that the CD55 S/T domain is necessary for nuclear localization offers a mechanism to disrupt CD55 movement to the nucleus. This is highly significant given the specificity of nuclear localization of CD55 in cancer cells. Immunoprecipitation of CD55 followed by mass spectrometry identified Tight Junction Protein 2 (TJP2) as an abundant and specific nCD55 binding protein (Fig. 6B). Our IP-MS studies indicate CD55-TJP2 interaction is strictly in the nucleus (Fig. 6B). Notably, TJP2 is found in the nucleus when maintained in cell cultures [56]. Our studies were performed in confluent cells, and we detected the interaction between CD55 and TJP2 in the nucleus. TJP2 was previously shown to impact the nuclear import of cytoplasmic proteins [57]. In the nucleus, TJP2 interacts with heterogeneous nuclear ribonucleoprotein scaffold attachment factor B [58]. TJP2 contains a PDZ phosphoinositide (PI) binding domain sufficient to localize phosphatidylinositol 4,5-bisphosphate (PtdIns(4,5)*P*_2_) to the nucleus in a speckled distribution pattern similar to the punctate nuclear distribution of nCD55 we observed [59]. Meerschaert and colleagues utilized PIP2 though they indicate that all PIs bind to TJP2. The findings implicate TJP2 as a candidate essential for recruitment of CD55 to the nucleus via binding to PDZ2 - PI moiety. Based on the identification of the lipid binding/PI domain within PDZ2, in future studies, we will investigate whether mutants in this region of TJP2 will disrupt the nuclear localization of CD55. This offers a potential strategy for disrupting nCD55 without suppressing the role of CD55 at the cell surface in the regulation of complement.

The CD55 S/T domain is also sufficient to bind to chromatin and lead to suppression of ZMYND8 protein expression (Figs. 4G and 6J). co-IP and mass spectrometry identified nCD55 complexes with the epigenetic regulator ZMYND8 in cancer cells. ZMYND8 was previously reported to be a tumor suppressor [45]. Similarly, we observed that loss of ZMYND8 increased cell proliferation, CSC self-renewal, and cisplatin resistance in OC cells (Fig. 6F, G, H, and I). Our studies show that ZMYND8 suppresses CD55 protein expression and CD55 inhibits ZMYND8 expression at the protein level, highlighting mutual reciprocal regulation of oncogenic CD55 and tumor suppressive ZMYND8. Moreover, the findings indicate that CD55 regulates ZMYND8 post-transcriptionally, suggesting that protein-protein interaction impacts protein stability.

The discovery of the CD55-ZMYND8 complex is particularly relevant as previous reports show that ZMYND8 complexes with EZH2 attenuate metastasis inducing genes [48]. We found that ZMYND8 was able to interact with EZH2 and SUZ12 in CD55 overexpressing cells. Epigenetic regulation of gene expression through modulating transcription is necessary for maintaining cellular functions, identity, and chromatin state in eukaryotic cells [60]. Our findings indicate that nCD55 binds to and suppresses ZMYND8 expression, thus derepressing PRC2 to increase H3K27Me3, potentially impacting CSCs and chemoresistance. Importantly, H3K27 is methylated by the PRC2 complex methylase EZH2 leading to global gene repression [61, 62] to promote cancer stem cell identity. Our findings suggest nCD55 may also regulate CSC identity through modulation of PRC2 activity.

We reveal a function for CD55 in the nucleus that is essential for resistance to cisplatin in pre-clinical studies. As in the in vitro analyses, the S/T domain phenocopies the effect of full-length CD55 in mice, suggesting this domain could be important for therapeutic targeting. In the cellular life cycle of CD55, its S/T domain is displayed on the cell surface, making it amenable to extracellular targeting with small molecule or antibody strategies [63]. Future studies will focus on the development of therapeutics via targeting of the S/T domain.

Our studies implicate nuclear CD55 as a marker of CSCs and chemoresistant disease. In cell-based studies, we find enrichment of CD55 in the nucleus of CSCs and chemoresistant cells. Moreover, in patient tumor specimens, nCD55 positive cancer cells are found in a patchy distribution suggesting localization in a CSC niche [64]. We also demonstrate that nCD55 is enriched in cells derived from ascitic fluid of chemoresistant OC patients further supporting a pathological role for nCD55 in cancer progression. In future studies, we will investigate whether nuclear CD55 is directly inducing metastasis via EZH2 [44, 48]. Finding nCD55 in patient tumor specimens may also provide insights into how ovarian cancer patients are diagnosed, risk-stratified, or evaluated in therapy. nCD55 is uniquely expressed in chemoresistant ascites offering a potential biomarker for treatment. The putative role of nCD55 as a biomarker may range from initial diagnosis to alternate treatment approaches for these patients that will require additional studies.

### Limitations of the study

Our studies identify CD55 is uniquely localized in the nucleus in ovarian cancer cells. We analyzed a battery of ovarian cancer lines and a PDX model and determined that chemoresistant lines consistently exhibit high nuclear CD55. While our studies focus on ovarian cancers, CD55 may localize in the nucleus of additional cancers and will be screened in the future. The specific mechanisms of CD55 nuclear entry and its impact on the epigenome remain unresolved leaving opportunities for further investigation. The reciprocal regulation of CD55 and ZMYND8 may provide insights into this question as CD55 may displace ZMYND8 from the PRC2 complex leading to CSC induction and chemoresistance. Our studies focused on nuclear CD55 impact on promoting CSCs and chemoresistance mechanisms. We recognize that CD55 may have an impact on other hallmarks of cancer cells including cell migration, metastasis/invasion, and angiogenesis. A related outstanding question is why CD55 is found in the nucleus of cancer cells but not in normal cells. Though unanswered, the enrichment of CD55 in ovarian CSCs may hold a key to this question. In future studies, we will investigate differential nCD55 in normal stem cells versus CSCs.

## Conclusions

We identify a new nuclear and chromatin-directed role for CD55 in chemoresistant ovarian cancers. Disruption of CD55 trafficking to the nucleus provides a molecular hook for targeting chemoresistant disease with the potential for reduced toxicity. The findings indicate that CD55, via its S/T domain, interacts with chromatin and complexes with epigenetic machinery promoting CSCs and chemoresistance (Fig. 8). The findings in cell culture are consistent with a role for nuclear CD55 as a potential biomarker of chemoresistant disease.

### Electronic supplementary material

Below is the link to the electronic supplementary material.

Supplementary Material 1

## Data Availability

Bulk RNA sequencing data is available through Zenodo at 10.5281/zenodo.10794409. Other data are available in supplementary figures. All uncropped western blots used in this study were shown in the supplementary material.

## Abbreviations

ANOVA: Analysis of Variance
BSA: Bovine serum albumin
CAS9: CRISPR associated protein 9
CD55: Complement decay-accelerating factor known as DAF
CRISPR: Clustered regularly interspaced short palindromic repeats
CSC: Cancer stem cells
DMEM: Dulbecco’s Modified Eagle Medium
EGF: Epidermal growth factor
ELDA: Extreme Limiting Dilution Analysis
EZH2: Enhancer of zeste homolog 2
FBS: Fetal bovine serum
FFPE: Formalin-Fixed Paraffin-Embedded
GFP: green fluorescent protein
GPI: Glycosylphosphatidylinositol
H3K27Me: Methylation of lysine 27 on histone H3
HGSOC: High-grade serous ovarian cancer
IHC: Immunohistochemistry
IVIS: In Vivo Imaging System
JAK: Janus kinase
JNK: c-Jun N-terminal kinases
KO: Knock out
LCK: lymphocyte-specific protein tyrosine kinase
LC-MS/MS: Liquid chromatography-tandem mass spectrometry
MAPK: Mitogen-activated protein kinases
Na3VO4: Sodium orthovanadate
NaCl: Sodium chloride
NF-KB: Nuclear factor kappa-light-chain-enhancer of activated B cells
NSG: NOD-scid IL2Rgamma^null^
OC: Ovarian cancer
PIPLC: Phosphatidylinositol-specific phospholipase C
PMSF: Phenylmethylsulfonyl fluoride
PRC2: Polycomb repressive complex 2
PVDF: Polyvinylidene difluoride
ROR2: Receptor Tyrosine Kinase Like Orphan Receptor 2
S/T: Serine and threonine rich domain
SCR: Short consensus repeats
SDS-PAGE: Sodium dodecyl sulfate–polyacrylamide gel electrophoresis
ShRNA: short hairpin RNA
STAT3: Signal transducer and activator of transcription 3
TBST: Tris-buffered saline with 0.1% Tween® 20 detergent
TJP2: Tight junction proteins 2
TUNEL: Terminal deoxynucleotidyl transferase dUTP nick end labeling
ZMYND8: Zinc finger MYND domain-containing protein 8
