# Supplementary material for: Cell surface CD55 traffics to the nucleus leading to cisplatin resistance and stemness by inducing PRC2 and H3K27 trimethylation on chromatin in ovarian cancer
Source: Mol Cancer. 2024 Jun 10;23:121. doi: 10.1186/s12943-024-02028-5 (PMC11163727; doi:10.1186/s12943-024-02028-5)

**Supplementary file**

**Cell Surface CD55 traffics to the nucleus leading to cisplatin resistance and Stemness by Inducing PRC2 and H3K27 trimethylation on Chromatin in Ovarian Cancer**

Rashmi Bharti^1#^, Goutam Dey^1#^, Debjit Khan^1^, Alex Myers^1^, Olivia G. Huffman^1^, Caner Saygin^1,+^, Chad Braley^1^, Elliott Richards^1,2^, Naseer Sangwan^1,3,4^, Belinda Willard^5^, Justin D. Lathia^1,4^, Paul L. Fox^1,4^, Feng Lin^4,6^, Babal Kant Jha^4,7^, J. Mark Brown^4,8^, Jennifer S. Yu^4,8^, Mohammed Dwidar^1,4,9^, Amy Joehlin-Price^10^, Roberto Vargas^4,11^, Chad M. Michener^4,11^, Michelle S. Longworth^4,6^, and Ofer Reizes^1,4^*

^1^Department of Cardiovascular and Metabolic Sciences, Lerner Research Institute, Cleveland Clinic Foundation, Cleveland, Ohio, USA

^2^Reproductive, Endocrinology, and Infertility, Obstetrics and Gynecology Institute, Cleveland Clinic Foundation, Cleveland, OH, USA

^3^Microbiome Analytics and Composition Core Facility, Lerner Research Institute, Cleveland Clinic Foundation, Cleveland, Ohio.

^4^Case Comprehensive Cancer Center, Cleveland, OH, USA

^5^Proteomics and Metabolomics Core, Lerner Research Institute, Cleveland Clinic, Cleveland, OH, 44106, USA

^6^Department of Immunity and Inflammation, Lerner Research Institute, Cleveland Clinic, OH, USA

^7^Center for Immunotherapy & Precision Immuno-oncology, Lerner Research Institute, Cleveland Clinic Foundation, Cleveland, Ohio, USA.

^8^Department of Cancer Biology, Lerner Research Institute of the Cleveland Clinic, Cleveland, Ohio, USA.

^9^ Microbial Culturing and Engineering Facility, Cleveland Clinic, Cleveland, Ohio, USA.

^10^ Anatomic Pathology, Pathology and Lab Medicine Institute, Cleveland Clinic Foundation, OH, USA.

^11^Department of Gynecologic Oncology, Obstetrics and Gynecologic Institute, Cleveland Clinic Foundation, Cleveland, Ohio, USA.

^+^Current address: Section of Hematology/Oncology, Department of Medicine, University of Chicago.

^#^Co-first authors

*Address correspondence to:

Ofer Reizes, Ph.D.

Department of Cardiovascular and Metabolic Sciences

Lerner Research Institute

9500 Euclid Avenue

Cleveland Clinic

Cleveland, OH 44195

Email: [reizeso@ccf.org](mailto:reizeso@ccf.org)

Direct: 216-445-0880

**Supplementary table1:** Reagents and resource

| REAGENT or RESOURCE | SOURCE | IDENTIFIER |
| --- | --- | --- |
| Antibodies |  |  |
| CD55 antibody | Proteintech (WB 1:3000, IHC: 1:500) | RRID:AB_2880559 Cat: 26580-1-AP |
| CD55 antibody | EMD Millipore (WB 1:400) | Cat: CBL511 |
| ZMND8 antibody | Proteintech (WB 1:1000) | RRID:AB_2241834 Cat: 11633-1-AP |
| CD59 antibody | Proteintech (WB 1:1000) | Cat: 10742-1-AP |
| AEBP2 antibody | Proteintech (WB 1:1000) | Cat: 11232-2-AP |
| EED antibody | Proteintech (WB 1:2000) | Cat: 16818-1-AP |
| SUZ12 antibody | Proteintech (WB 1:2000) | Cat: 20366-1-AP |
| EZH1 antibody | Proteintech (WB 1:1000) | Cat: 20852-1-AP |
| EZH2 antibody | Proteintech (WB 1:5000) | Cat: 21800-1-AP |
| JRID2 antibody | Cell signaling (WB 1:1000) | Cat: D6M9X |
| Ki-67 antibody | Proteintech (IHC 1:5000) | Cat: 27309-1-AP |
| CD55 antibody | EMD millipore (IHC 1:500) | Cat: CBL511 |
| Na^+^ K^+^ ATPase antibody | Cell Signaling (WB 1:3000) | RRID:AB_2060983 Cat: 3010S |
| H3 antibody | Proteintech (WB 1:3000) | RRID:AB_2716755 Cat: 17168-1-AP |
| H3K4Me3 antibody | EMD Millipore (WB 1:3000) | RRID:AB_1977252 Cat:07-473 |
| GAPDH antibody | Proteintech (WB 1:3000) | Cat: HRP-60004 RRID:AB_2737588 |
| Lamin A/C antibody | Proteintech (WB 1:3000) | Cat:10298-1-AP RRID:AB_2296961 |
| Alpha Tubulin Monoclonal antibody | Proteintech (WB 1:3000) | Cat: HRP-66031 RRID:AB_2687491 |
| Mouse IgG XP® Isotype Control antibody | Cell signaling | Cat: 5415S RRID:AB_10829607 |
| Rabbit IgG XP® Isotype Control antibody | Cell signaling | Cat: 3900S RRID:AB_1550038 |
| Anti-Mouse IgG (H+L), HRP antibody | Promega (WB 1:30000) | Cat: W4028 |
| Anti-Rabbit IgG (H+L), HRP antibody | Promega (WB 1:30000) | Cat: W4018 |
| Goat anti-mouse IgG Alexa Fluor 568 | Thermo (IFC 1: 1000) | Cat: A11031 RRID:AB_144696 |
| Chemicals and reagents |  |  |
| Immobilon-P Membrane | Merck Millipore | Cat: IPVH00010 |
| Epidermal growth factor | R and D system | Cat: 236-EG-200 |
| B-27 Supplement | Gibco | Cat: 17504044 |
| Fibroblast growth factor | Gibco | Cat: 100-18B-1MG |
| Precision Plus Protein™ Kaleidoscope | Biorad | Cat: 1610375 |
| SYBR™ Gold Nucleic Acid Gel Stain | Thermo | Cat: S11494 |
| Cisplatin | Fesenius Kabi | Cat: 401572I |
| Pierce™ Protein A/G Agarose | Thermo | Cat: 20423 |
| Laemmli sample buffer, reducing (6X) | Alfa Aesar | Cat: J61337 |
| Trypan Blue | Fisher scientific | Cat: 25900CI |
| Critical Commercial Assays |  |  |
| NP40 Lysis buffer | Thermo | Cat: FNN0021 |
| RNA isolation kit | Takara | Cat: 740984.250 |
| ImmPRESS Goat ant-mouse IgG Polymer Kir, Peroxidase | Vector lab | Cat: MP-7452 |
| ImmPACT^TM^ DAB | Vector lab | Cat: SK-4105 |
| Protease Inhibitor Cocktail | Sigma | Cat: 04693159001 |
| NuPAGE™ Protein Gel | Thermo | Cat: NP0329BOX |
| 4–20% Mini-PROTEAN Protein Gels | Biorad | Cat: 4568096 |
| TUNEL Kit | Sigma | Cat:11684795910 |
| pcDNA™3.1 Directional TOPO™ Expression Kit | Thermo | Cat: K490001 |
| One Shot™ Stbl3™ Chemically Competent E. coli | Thermo | Cat: C737303 |
| Gateway™ LR Clonase™ Enzyme mix | Thermo | Cat: 11791019 |
| Pierce BCA Protein Assay Kit | Thermo | Cat: 23225 |
| Cytoplasmic and nuclear protein isolation kit | Thermo | Cat: 78835 |
| Subcellular protein fraction kit | Thermo | Cat: 78840 |
| Cyclohexamide | Selleckchem | Cat: S7418 |
| Histone extraction kit | Epigentek | OP-0006-100 |
| Pierce™ IP Lysis Buffer | Thermo | Cat: 87788 |
| CellTiter-Glo® 2.0 Cell Viability Assay | Promega | Cat: G9241 |
| Lipofectamin 3000 | Thermo | Cat: L3000001 |
| Live/Dead assay kit | Thermo | Cat: L23105 |
| VECTASHIELD^®^ Mounting Medium | Vector lab | Cat: H-1200 |
| PIPLC enzyme | Thermo | Cat: P-6466 |
| Recombinant DNA |  |  |
| pLenti CMV Puro DEST | Addgene | NA |
| CD55 WT pLenti CMV Puro DEST | In-house | NA |
| CD55 Δ1 pLenti CMV Puro DEST | In-house | NA |
| CD55 Δ2 pLenti CMV Puro DEST | In-house | NA |
| CD55 Δ3 pLenti CMV Puro DEST | In-house | NA |
| CD55 Δ4 pLenti CMV Puro DEST | In-house | NA |
| CD55 Δ1234 pLenti CMV Puro DEST | In-house | NA |
| CD55 ΔST pLenti CMV Puro DEST | In-house | NA |
| CD55 Δ34ST pLenti CMV Puro DEST | In-house | NA |
| CD55 CRISPR/Cas9 KO Plasmid | Santa Cruz | sc-400738 |
| ZMYND8 CRISPR/Cas9 KO Plasmid | Santa Cruz | sc-411774 |
| Experimental Models: Cell Lines |  |  |
| HEK293: Human Embryonic Kidney cells | ATCC | NA |
| CP70: Ovarian epithelial carcinoma | Dr. Analisa Difeo | NA |
| SKOV3: Ovarian adenocarcinoma | ATCC | NA |
| Jurkat: Immortalized T lymphocyte | ATCC | NA |
| A2780: Ovarian epithelial carcinoma | Dr. Analisa Difeo | NA |
| T0V112D: Ovarian epithelial carcinoma | ATCC | NA |
| OV81: High-Grade Serous Carcinoma | Dr. Analisa Difeo | NA |
| OVCAR8: High-Grade Serous Carcinoma | Dr. Analisa Difeo | NA |
| Experimental model: Mouse |  |  |
| NOD.Cg*-Prkdc^scid^ Il2rg^tm1Wjl^/*SzJ (NSG) mice | Jackson Lab |  |
| Software |  |  |
| Graph Pad prism | www.graphpad.com | NA |
| ImageJ | imagej.nih.gov | NA |
| FlowJo | BD Bioscience | NA |

**Supplementary table 2**: Clinical details of the ovarian tumor specimen.
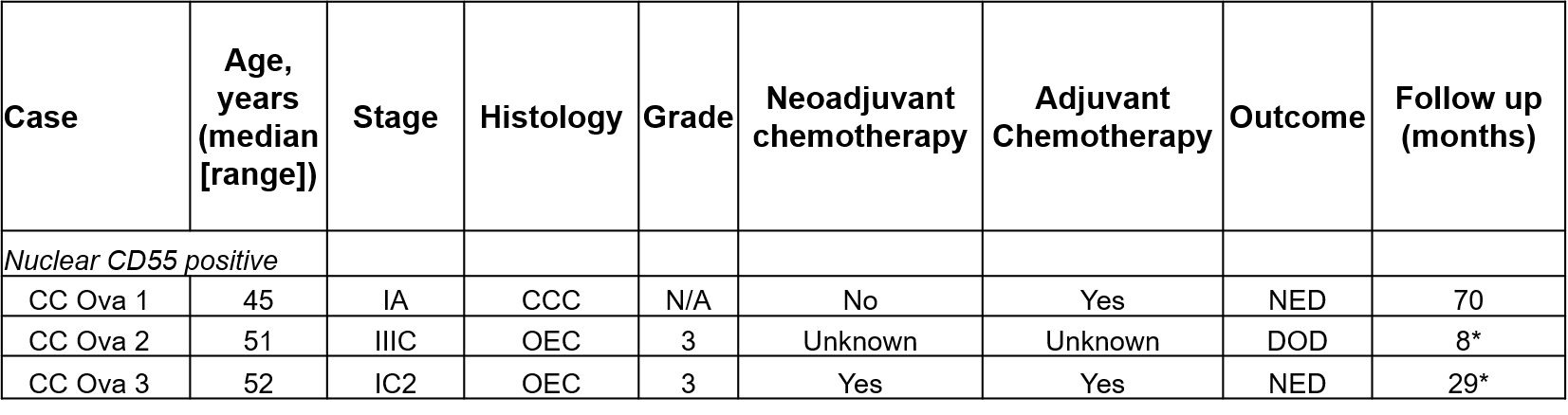


* Deceased

NED, no evidence of disease

DOD, dead of disease

CCC, Clear cell carcinoma

OEC, Ovarian endometrioid carcinoma

**Supplementary table 3:** Clinical details of the patients from where ascites cells were collected.

| Case | Age,  Years  (median [range]) | Stage | Histology | Grade | Adjuvant Chemotherapy | Chemoresistant | Outcome (recurrence year post treatment) |
| --- | --- | --- | --- | --- | --- | --- | --- |
| CCFOC45 | 62 years | IVa | High grade serous | high | Y | Y | 3 |
| CCF OC61 | 62 years | IIIc | High grade serous | high | Y | Y | 2 |
| CCF OC88 | 77 years | IIIc | High grade carcinoma | High  40% ER+ | Y | Y | 1 |

**Supplementary table 4:** Structure of CD55 protein and its domain deletion mutants.

| **Constructs** | **Description** |
| --- | --- |
| Wild type CD55 | Full length CD55 protein.  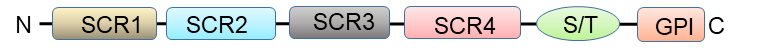 |
| Δ1 | SCR-1 domain deletion.  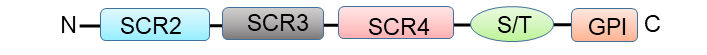 |
| Δ2 | SCR-2 domain domain deletion.  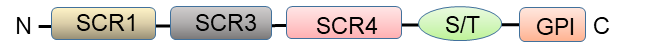 |
| Δ3 | SCR-3 domain deletion.  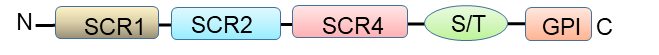 |
| Δ4 | SCR-4 domain deletion.  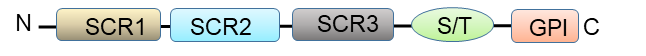 |
| Δ1234 | SCR-1, SCR-2, SCR-3, and SCR-4 domains deleted. Mutant contains only Serine/Threonine (S/T) domain.  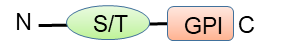 |
| ΔST | Serine/Threonine (ST) domain deletion.  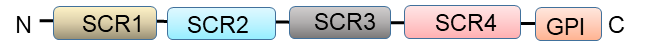 |
| Δ34 | SCR-3 and SCR-4 domains deleted.  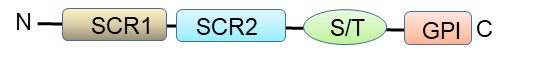 |
| Δ34ST | SCR-3 and SCR-4 as well as Serine/Threonine (ST) domain deleted.  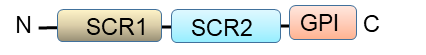 |

**Supplementary table 5:** List of primers used in CD55 mutagenesis.

| **CD55 mutants** | **Primers** |
| --- | --- |
| CD55 Wild type | Forward: CACCGCCGCCATGACCG  Reverse: CTAAGTCAGCAAGCCCATGG |
| CD55∆1 | Forward: AAGATTTGAGCTGCGAGGTGCCAACA  Reverse: CGCAGCTCAAATCTTCTTCAGAAATCAACTTT |
| CD55∆2 | Forward: GCAATCGTAAATCATGCCCTAATCCGGGAGA  Reverse: ATGATTTACGATTGCAGAACTCTTCAATATCT |
| CD55∆3 | Forward: GTAAAAAGATTTATTGTCCAGCACCACCAC  Reverse: AATAAATCTTTTTACAAAATTCGACTGCTGTG |
| CD55∆4 | Forward: GCAGAGAAAAATCTCTAACTTCCAAGGTCCC  Reverse: GAGATTTTTCTCTGCACTCTGGCAACG |
| CD55∆1234 | Forward: AAGATTTGAAATCTCTAACTTCCAAGGTCCC  Reverse: GAGATTTCAAATCTTCTTCAGAAATCAACTTT |
| CD55∆ST | Forward: GCAGAGGACGTCTTCTATCTGGGCACACG  Reverse: GAAGACGTCCTCTGCATTCAGGTGGTGG |
| CD55∆34 | Forward: GTAAAAAGAAATCTCTAACTTCCAAGGTCCCAC  Reverse: GAGATTTCTTTTTACAAAATTCGACTGCTGTG |
| CD55∆34ST | Forward: GTAAAAAGCGTCTTCTATCTGGGCACACG  Reverse: GAAGACGCTTTTTACAAAATTCGACTGCTGTG |
|  |  |

**Supplementary table 6:** List of primers used in qRT-PCR

| **Gene** | **Primers** |
| --- | --- |
| ZMYND8 | Forward: CCAAAGCCCTTCTCTCCTCA  Reverse: CGCTCAGCTCCTTCAAATCC |
| GAPDH | Forward: TGTCAAGCTCATTTCCTGGTAT  Reverse: CTCTCTTCCTCTTGTGCTCTTG |


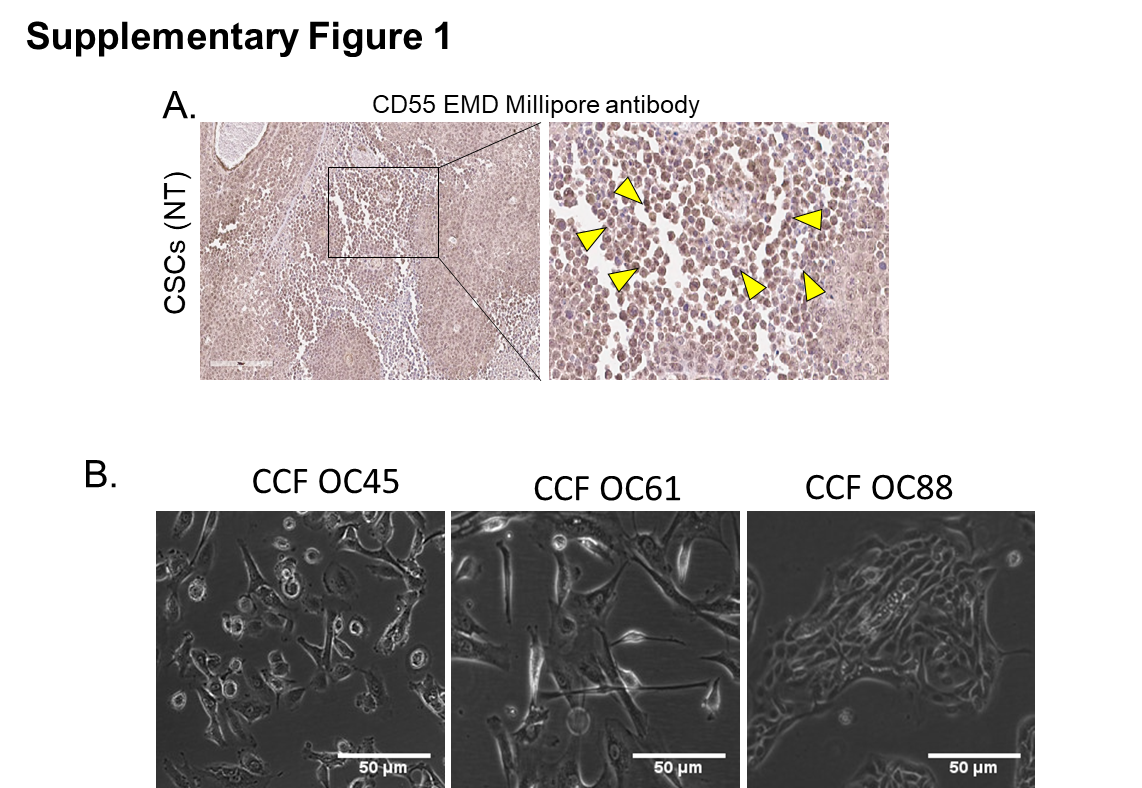


**Supplemental Fig. 1**

**(A)** Tumor specimen from A2780 CSCs non-targeted control (NT) was processed for immunohistochemistry using CD55 EMD Millipore antibody.

**(B)** Bright field microscopy to visualize morphology of the ascites cells. Images were captured at 10x magnification.


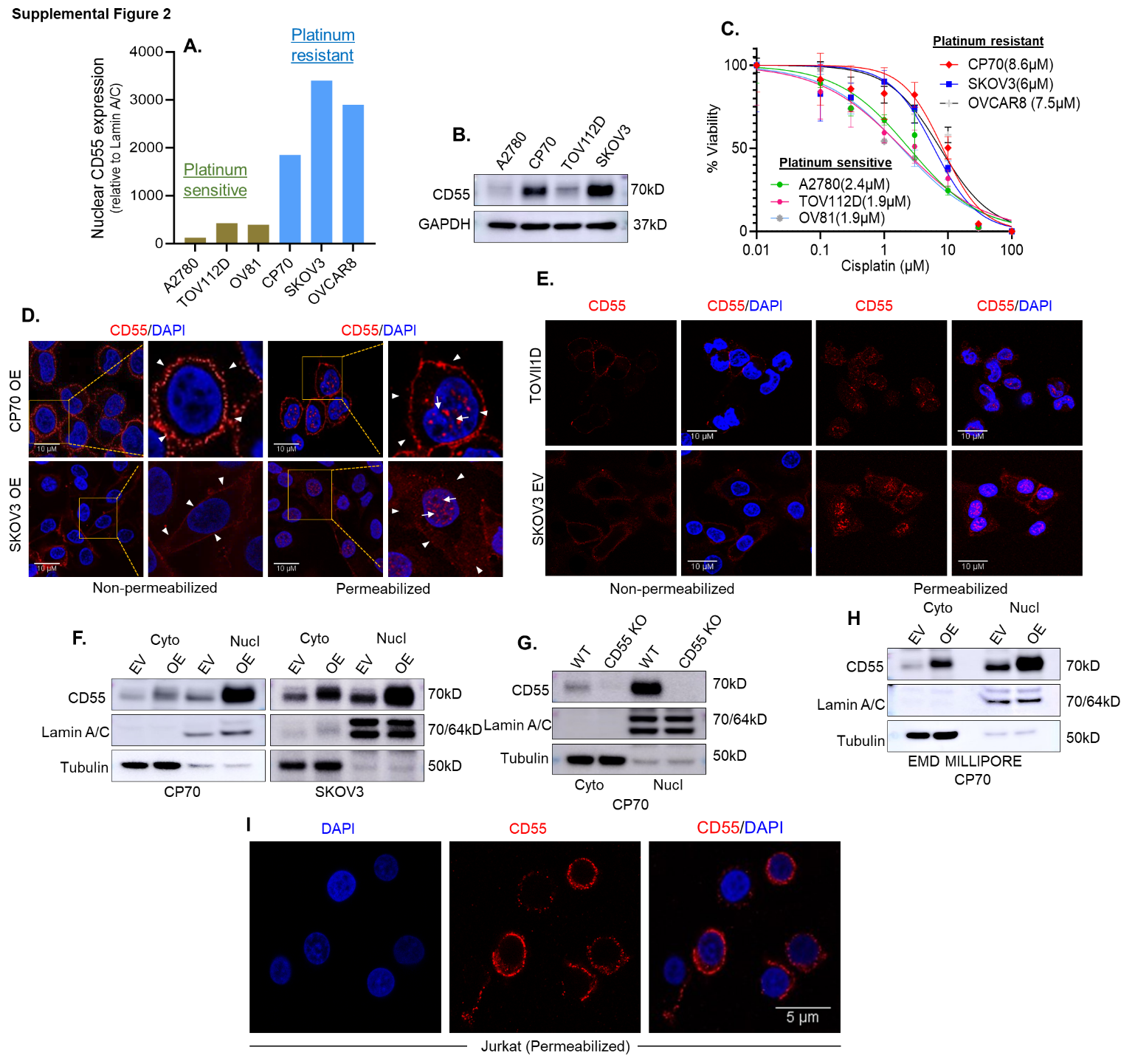


**Supplementary Fig. 2**

**(A)** Quantification of nuclear CD55 protein expression from Fig. 2B, C.

**(B)** CD55 protein expression in ovarian cancer cells, A2780, CP70, TOV112D, and SKOV3 cells.

**(C)** Platinum resistant (CP70, SKOV3 and OVCAR8) and platinum sensitive (A2780, TOV112D, and OV81) ovarian cancercells were treated with cisplatin for 48h. Following that cells were treated cell titre glow reagents to detect the cell viability. Data was analyzed and % of cell viability was measured and plotted in the graph. IC_50_ values of cisplatin were shown in the figure.

**(D)** Immunofluorescence staining of CP70 CD55 OE and SKOV3 CD55 OE cells under permeabilized (Using Triton X-100 during processing) or non-permeabilized conditions (No Triton X-100 during processing). Localization of CD55 (Red) was shown by arrowhead (Cytoplasmic) and arrow (Nuclear).

**(E)** Immunofluorescence staining of TOV112D and SKOV3 EV cells under permeabilized (Using Triton X-100 during processing) or non-permeabilized conditions (No Triton X-100 during processing).

**(F)** CP70 and SKOV3 cells transduced with empty vector or CD55 overexpression vector were processed for immunoblot analysis to evaluate cytoplasmic and nuclear expression of CD55 protein using Proteintech antibody.

**(G)** CP70 parental (Wild type, WT) and CP70 CD55 CRISPR knock out cells were subjected to cytoplasmic/nuclear fractionation and immunoblot study was conducted. Lamin AC was used as nuclear marker and Tubulin was used as cytoplasmic marker.

**(H)** Validation of nuclear CD55 protein in CP70 cells (EV and CD55 OE) by immunoblot experiment using EMD Millipore CD55 antibody.

**(I)** Jurkat cells were grown, fixed/permeabilized and immunofluorescence study was performed to visualize CD55 localization. Images were captured by confocal microscope at X63 magnification. CD55 protein localization was shown in red and nucleus was counter stained by DAPI (Blue).


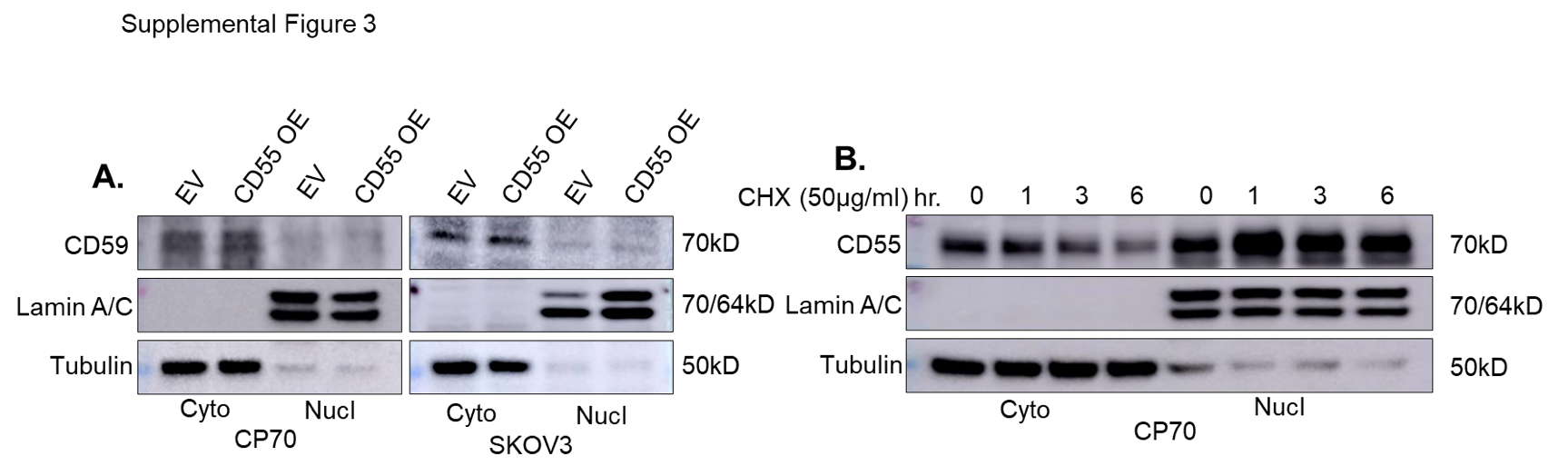


**Supplementary Fig 3**

**(A)** CP70 and SKOV3 cells transduced with empty vector or CD55 overexpression vector were processed for immunoblot analysis to evaluate cytoplasmic and nuclear expression of CD59 protein.

**(B)** CP70 cells were treated with cycloheximide (50µg/ml) for 0, 1, 3, and 6 hours, followed by cell fractionation for cytoplasmic and nuclear isolation. Samples separated on SDS-PAGE followed by western blotting for CD55 protein expression corrected to 0 time point. Quantification was shown in main Fig. 2G.


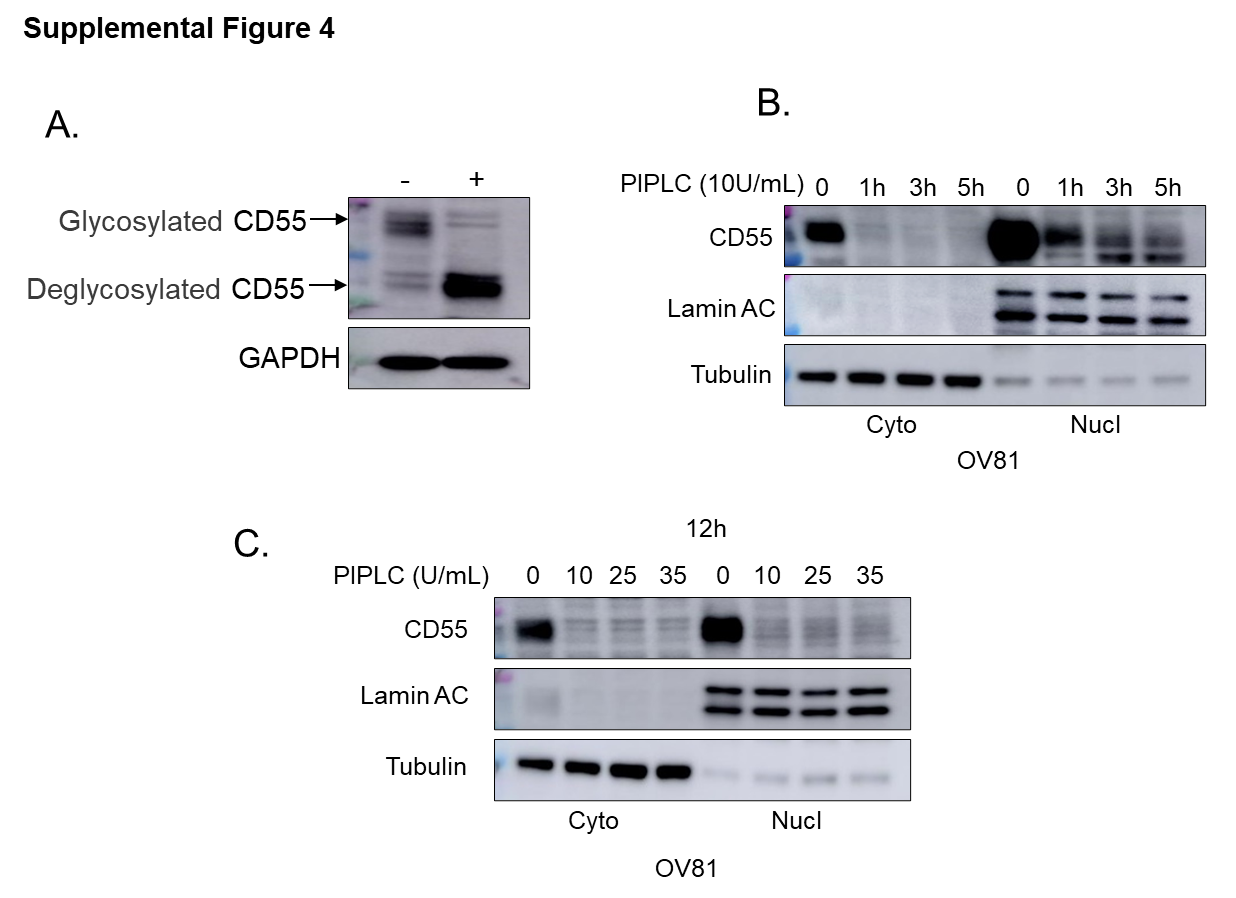


**Supplementary Fig. 4**

**(A)** CP70 ovarian cancer cells were lysed. The lysed proteins were then treated with protein deglycosylation mix II enzyme to remove glycosylation from CD55 proteins. The protein samples were processed for immunoblot analysis.

**(B)** OV81 ovarian cancer cells were treated with 10U/ml of PIPLC at 37°C for 1, 3, and 5 hours. Following cell harvesting, cytoplasmic and nuclear proteins were fractionated. Then, an immunoblot study was performed to check CD55 protein expression. Lamin A/C was used as nuclear protein loading marker and Tubulin was used as cytoplasmic loading control marker.

**(C)** OV81 ovarian cancer cells were treated with PIPLC (0, 10, 25 and 35U/mL for 12h). Following cell harvesting, cytoplasmic and nuclear proteins were fractionated. Then, an immunoblot study was performed to check CD55 protein expression. Lamin A/C was used as nuclear protein loading marker and Tubulin was used as cytoplasmic loading control marker.


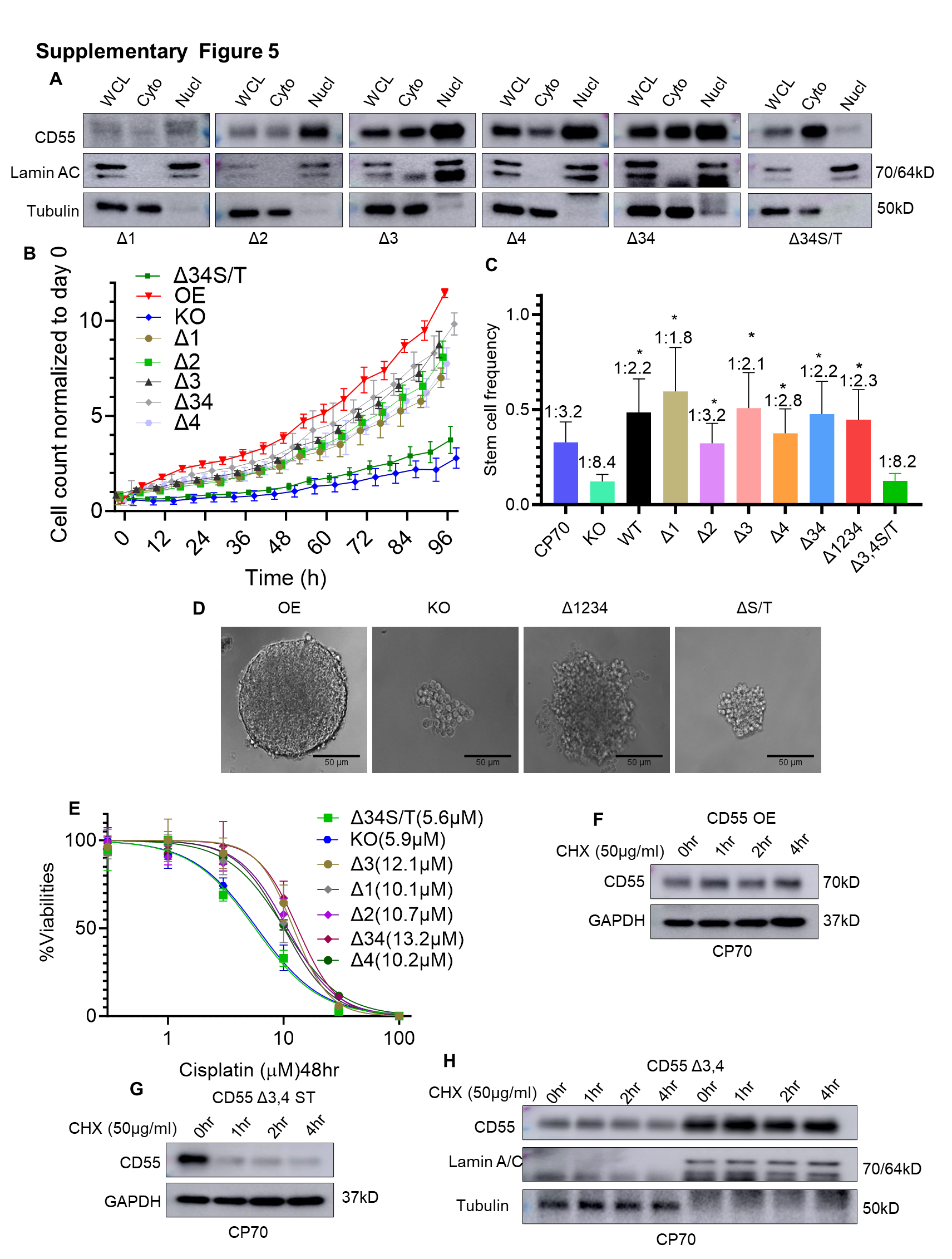


**Supplementary Fig. 5**

(A) CD55 domain deletion mutants were generated and introduced in CP70 CD55 knock out cells. CP70 CD55∆1, CP70 CD55∆2, CP70 CD55∆3, CP70 CD55∆4, CP70 CD55∆34, and CP70 CD55∆34ST were grown in 100mm Petri Dishes. Cells were collected and whole cell lysate (WCL), cytoplasmic, and nuclear proteins were prepared and immunoblot study was performed to check CD55 protein expression in each fraction.

(B) Cell proliferation analysis using Incucyte. CP70 CD55 KO, CP70 CD55 OE, CP70 CD55∆1, CP70 CD55∆2, CP70 CD55∆3, CP70 CD55∆4, and CP70 CD55∆34, were analyzed over a 4-day observation period. The data for CD55 OE and KO also was shown in main fig. 4D.

(C) Stem Cell frequency was analyzed by limiting dilution assay. One way ANOVA was performed, and Tukey’s multiple comparison test was performed to determine p values (* p < 0.05, ** p < 0.01, *** p < 0.001).

(D) Representative images of spheroids from CD55 OE, KO, ∆1234, and ∆S/T plated as single cells and monitored for 14 days from Fig.4E.

(E) Cisplatin sensitivity assay of CP70 CD55 KO, CP70 CD55∆1, CP70 CD55∆2, CP70 CD55∆3, CP70 CD55∆4, and CP70 CD55∆34. Data was analyzed with Graphpad Prism and IC_50_ values of cisplatin were indicated in parentheses.

(F) CP70 CD55 OE cells were treated with cycloheximide (50µg/ml) for 0, 1, 2, and 4h. Following that cells were collected and immunoblot study was performed to check CD55 protein expression.

(G) CP70 CD55∆34ST cells were treated with cycloheximide (50µg/ml) for 0, 1, 2, and 4h. Following that cells were collected and immunoblot study was performed to check CD55 protein expression.

(H) CP70 CD55∆34 cells were grown, and cells were collected. Then cytoplasmic and nuclear, protein fractions were prepared. CD55 protein expression was checked in each fraction by immune blot analysis.


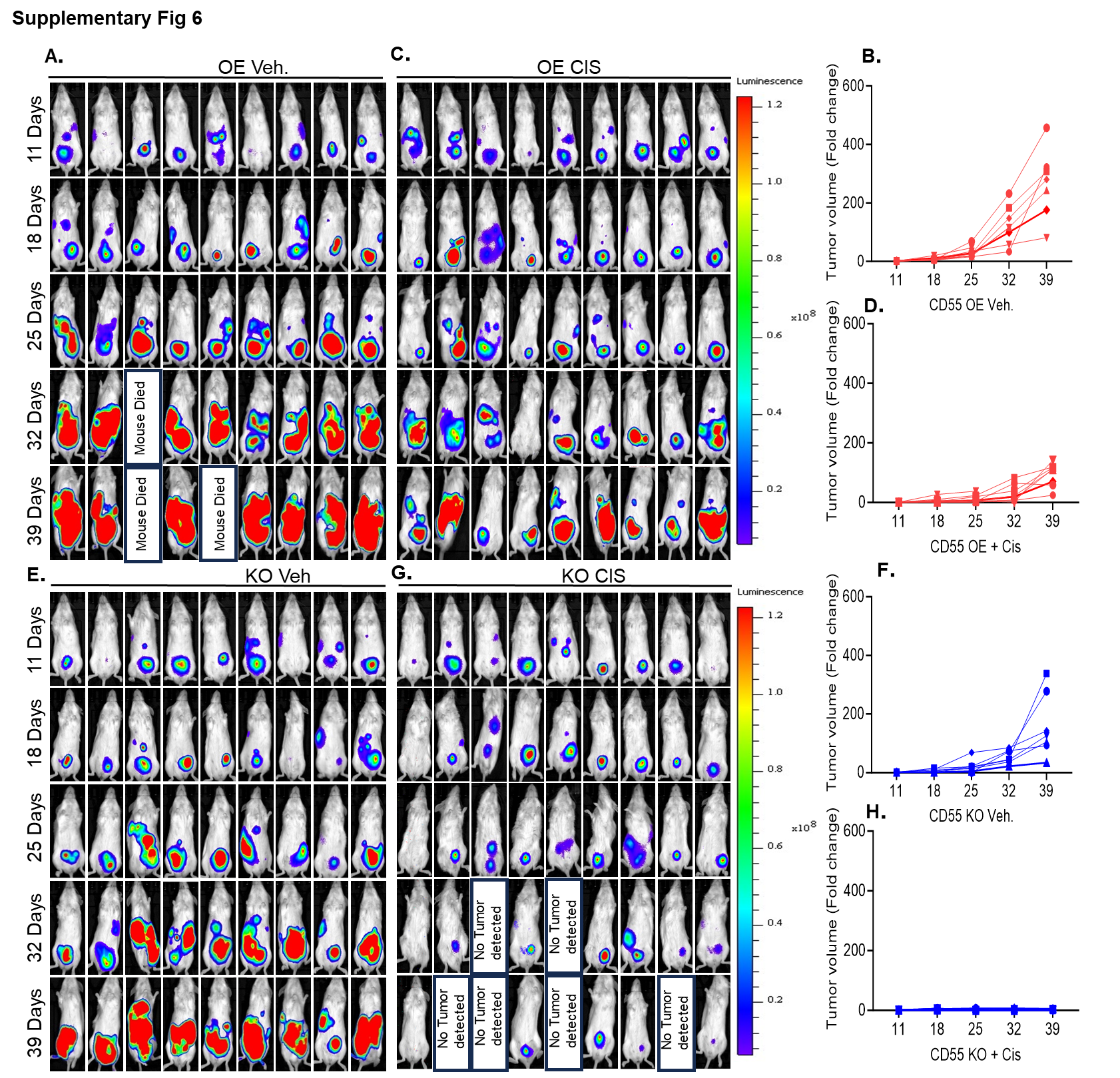


**Supplementary Fig. 6** *In Vivo* experiment in NOD-scid IL2Rgamma^null^ (NSG) mice. Mice were injected CP70 CD55 OE and CP70 CD55 KO ovarian cancer cells. After tumor development mice were treated with Cisplatin (2mg/kg twice/week) or Vehicle. During the study, mice were injected with D-Luciferin and bioluminescence images of the tumor in each mouse were captured by IVIS Lumina. Representative images of the mice were shown in the figure.

(A, B) IVIS images of tumor of each mouse from CD55 OE Veh cohort and tumor growth kinetics of individual mouse at different time interval.

(C, D) IVIS images of tumor of each mouse from CD55 OE Cis cohort and tumor growth kinetics of individual mouse at different time interval.

(E, F) IVIS images of tumor of each mouse from CD55 KO Veh cohort and tumor growth kinetics of individual mouse at different time interval.

(G, H) IVIS images of tumor of each mouse from CD55 KO Cis cohort and tumor growth kinetics of individual mouse at different time interval. Luminescence scale of the tumor images was shown.


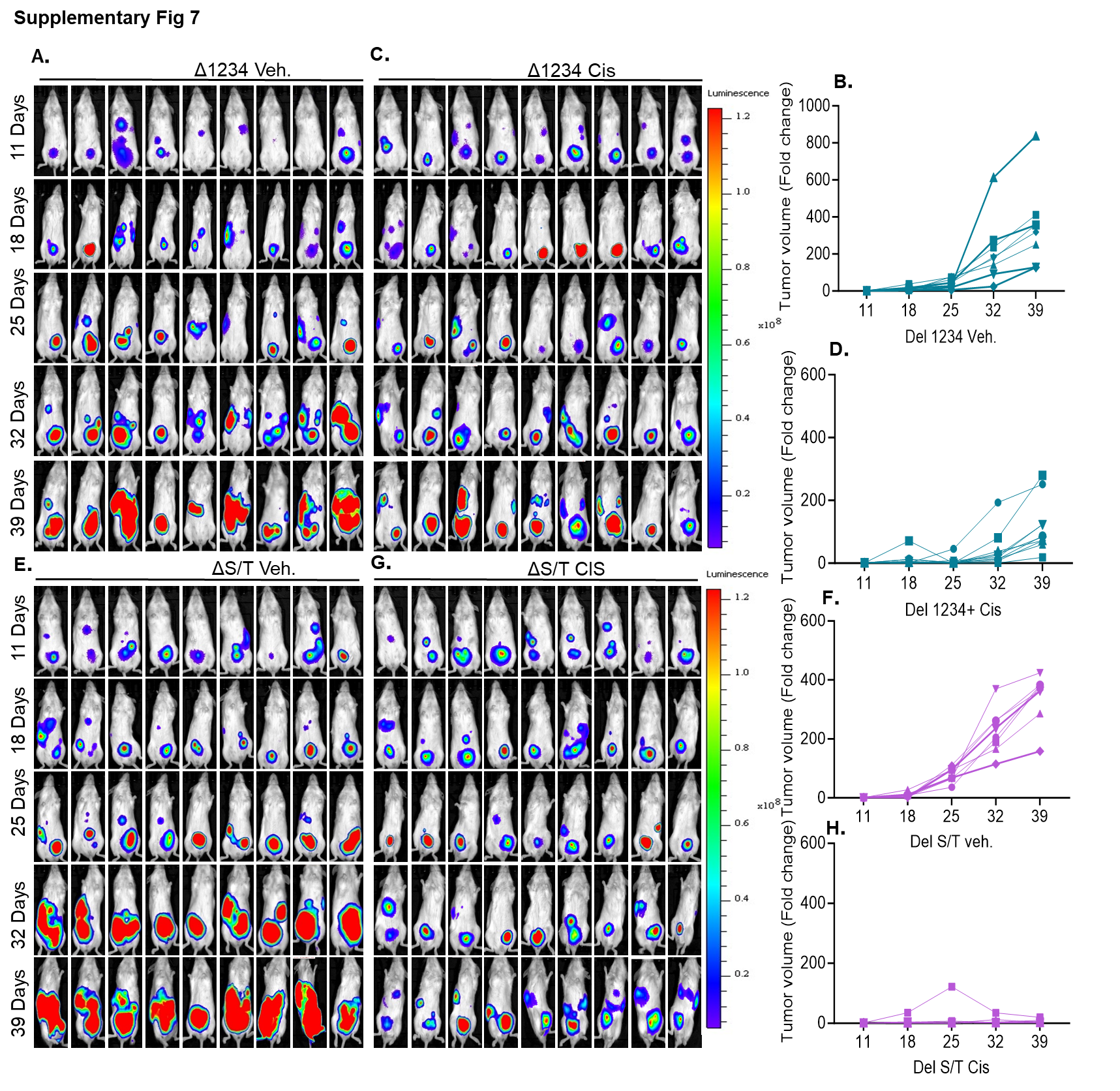


**Supplementary Fig. 7** In Vivo experiments in NOD-scid IL2Rgamma^null^ (NSG) mice. Mice were injected CP70 CD55∆1234 and CP70 CD55∆ST ovarian cancer cells. After tumor development mice were treated with Cisplatin (2mg/kg twice/week) or Vehicle. During the study, mice were injected with D-Luciferin and bioluminescence images of the tumor in each mouse were captured by IVIS Lumina.

**(A, B)** IVIS images of tumor of each mouse from CD55∆1234 Veh cohort and tumor growth kinetics of individual mouse at different time interval.

**(C, D)** IVIS images of tumor of each mouse from CD55∆1234 Cis cohort and tumor growth kinetics of individual mouse at different time interval.

**(E, F)** IVIS images of tumor of each mouse from CD55 ∆ST Veh cohort and tumor growth kinetics of individual mouse at different time interval.

**(G, H)** IVIS images of tumor of each mouse from CD55 ∆ST Cis cohort and tumor growth kinetics of individual mouse at different time interval. Luminescence scale of the tumor images was shown.


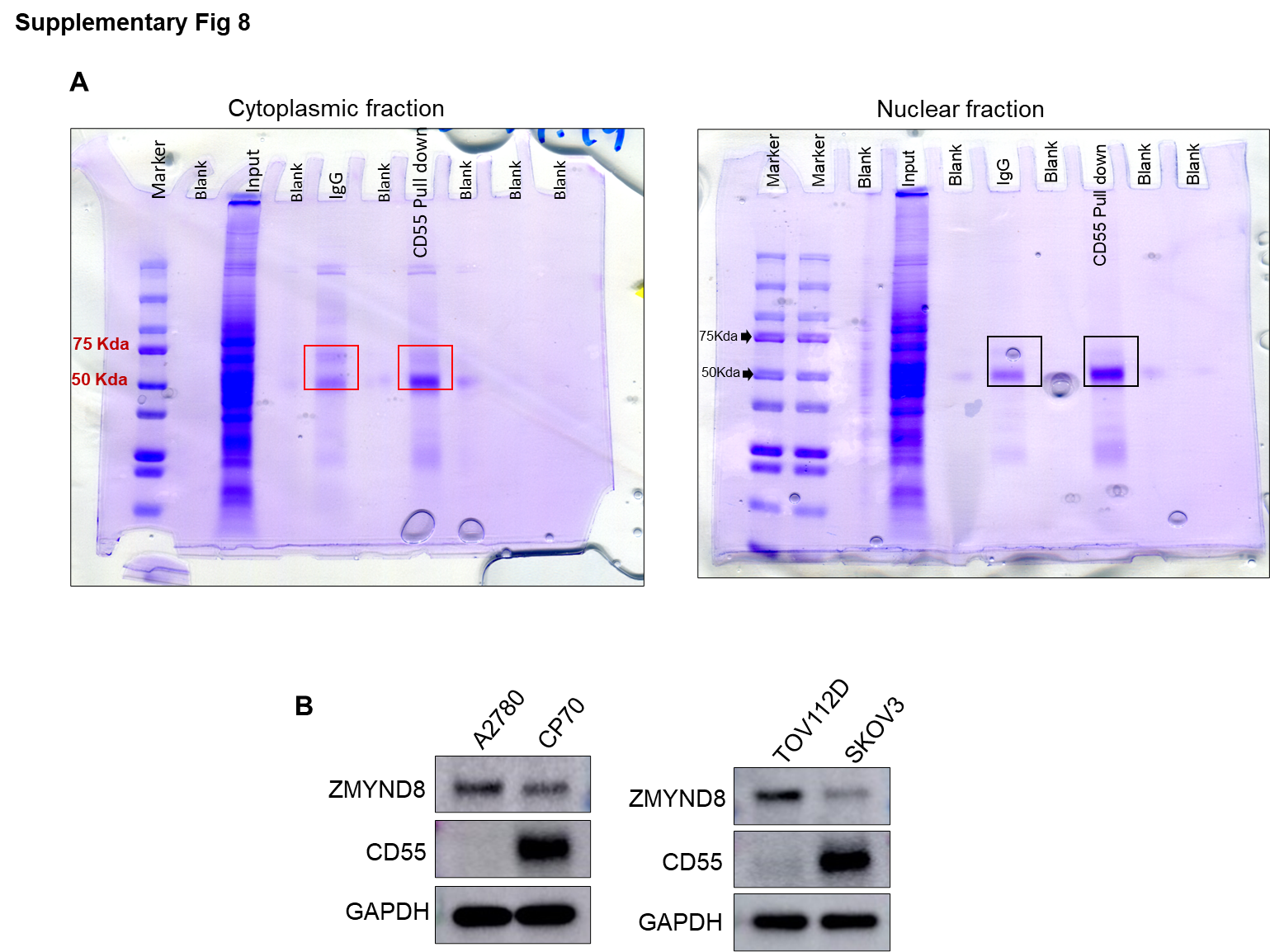


**Supplementary Fig. 8**

1. CD55 was immunoprecipitated from cytoplasmic and nuclear fractions of CP70, resolved by SDS-PAGE, followed by LCMS analysis. LCMS data was shown in main Fig. 6A, B.
2. Expression of ZMYND8 and CD55 proteins in sensitive vs resistant cells. CP70 and SKOV3 cells are platinum resistant cells where as A2780 and TOV112D cells are platinum sensitive cells.

**Whole blot images**


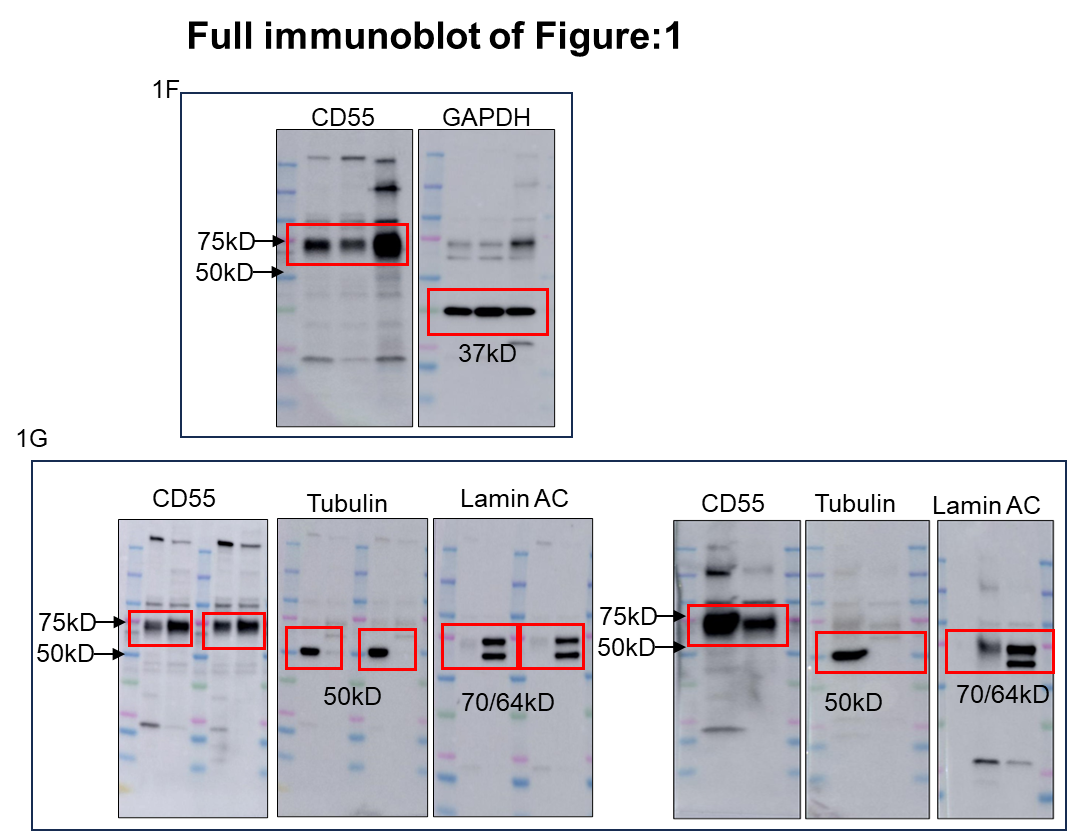


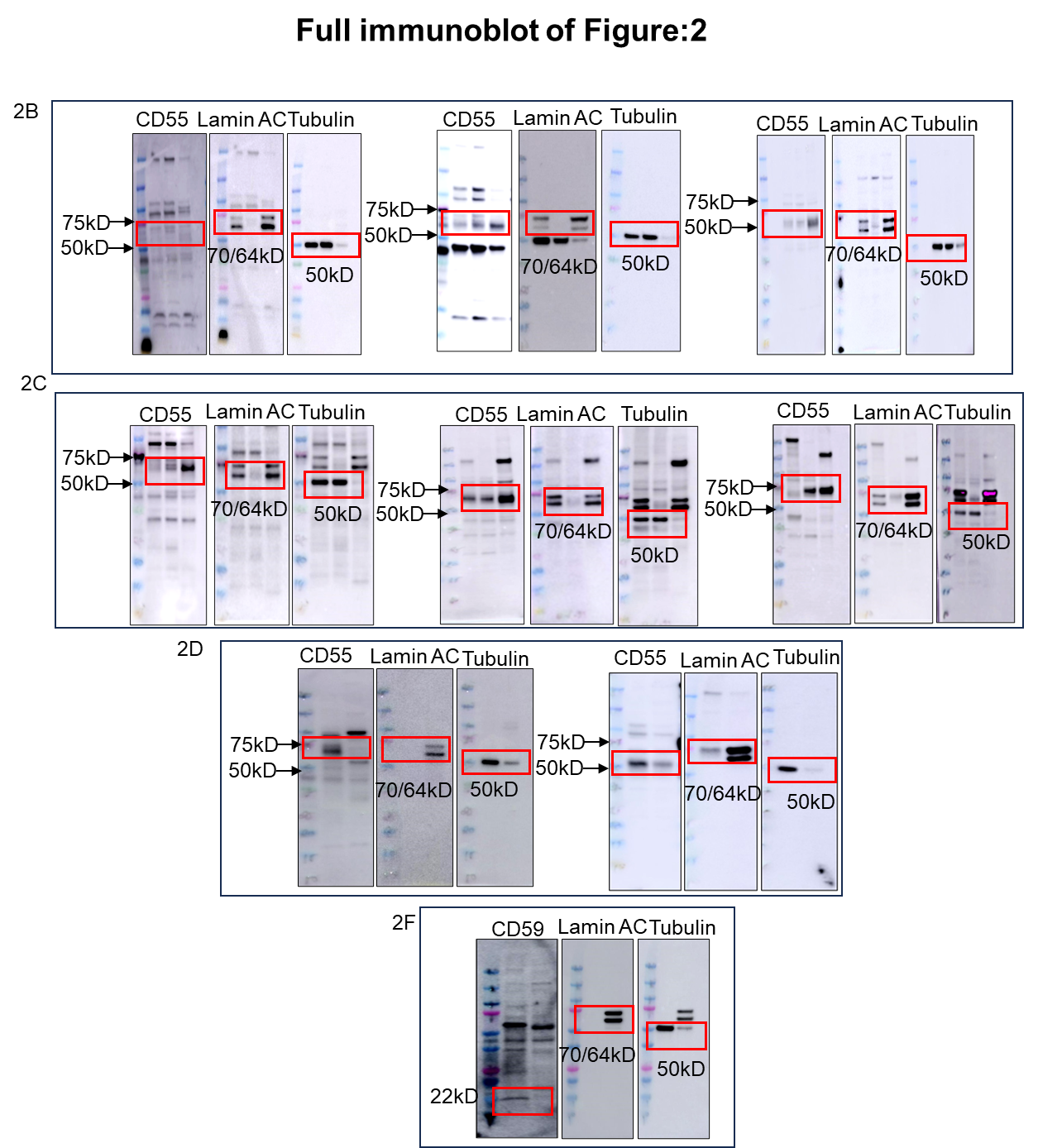


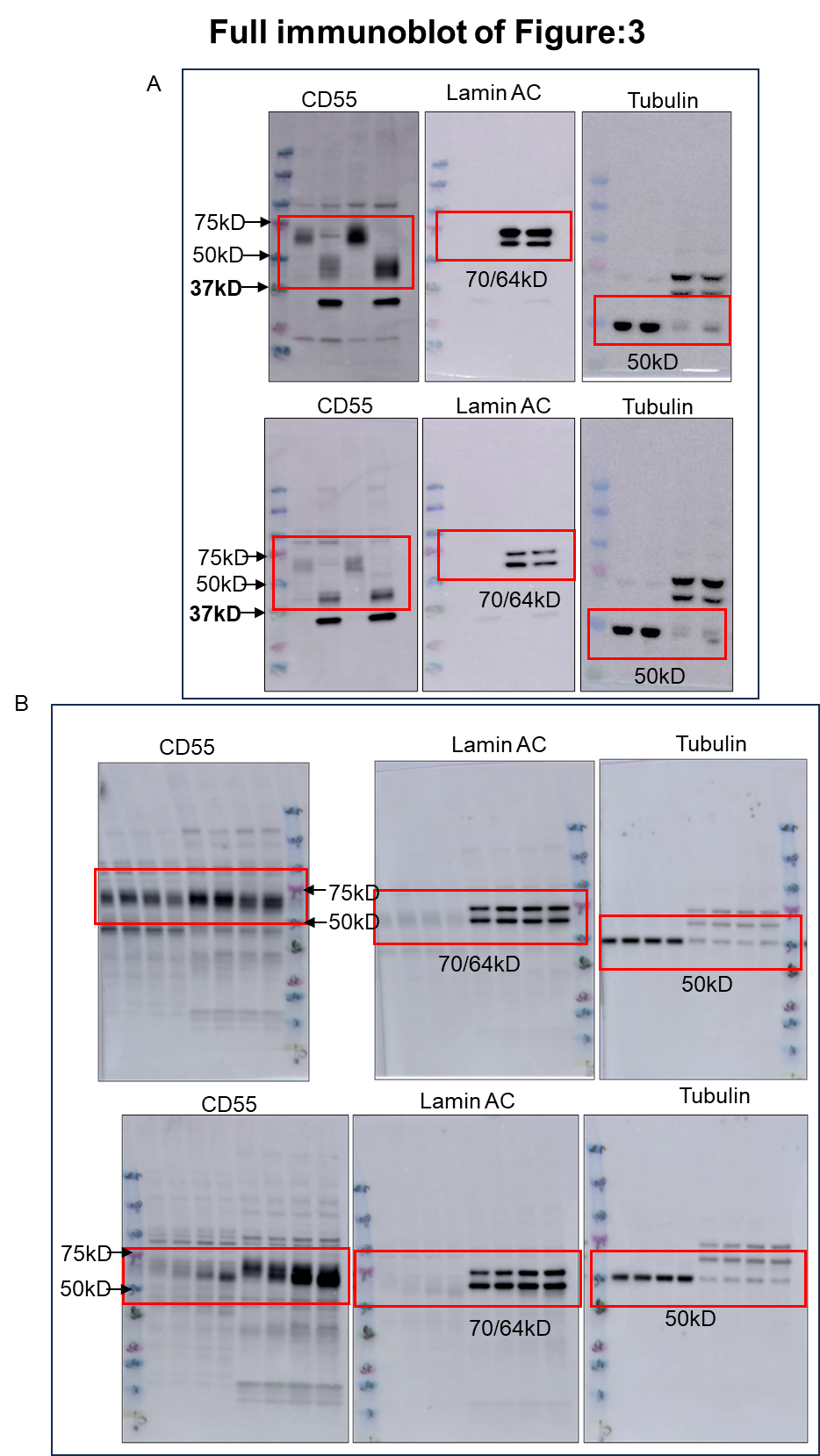


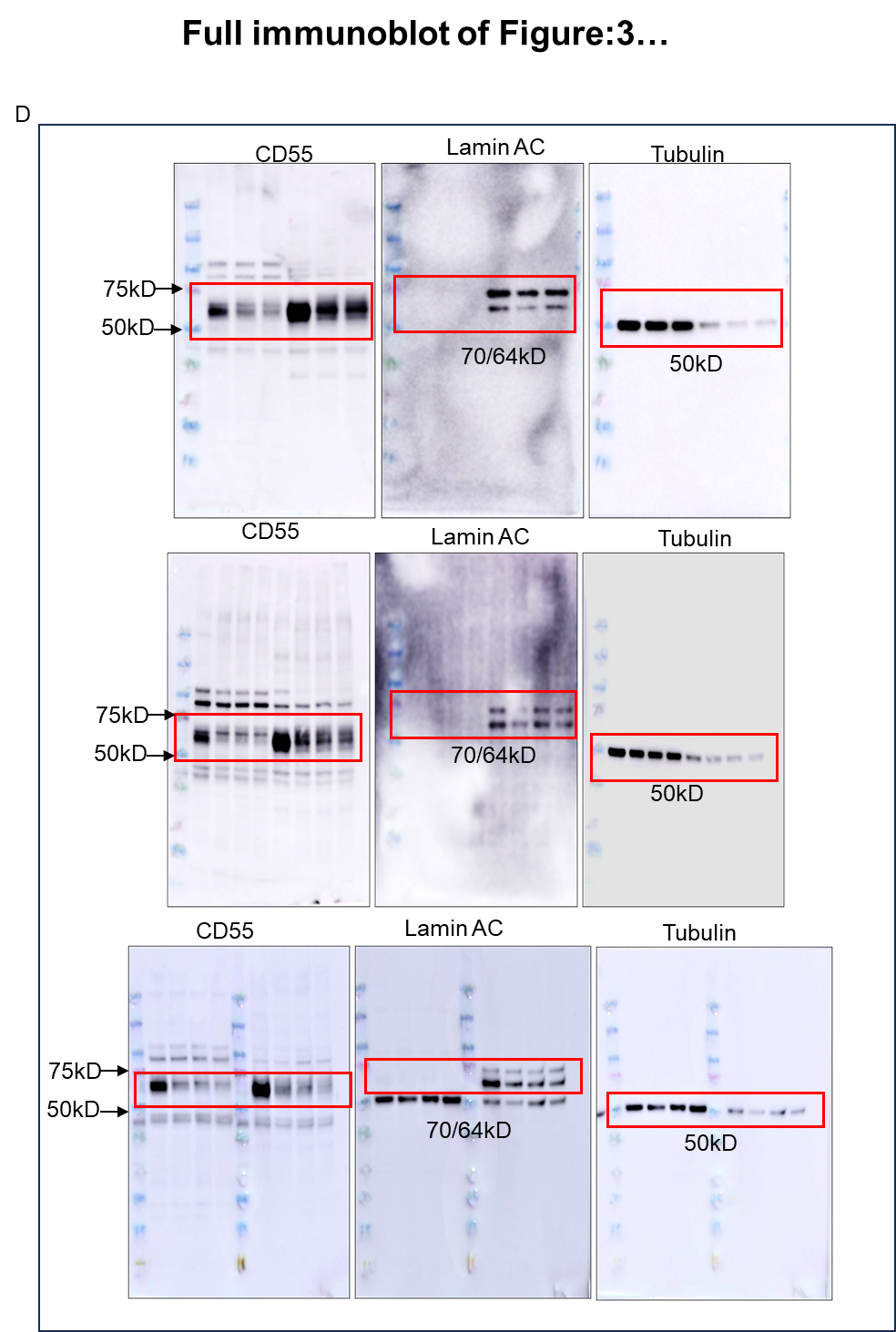


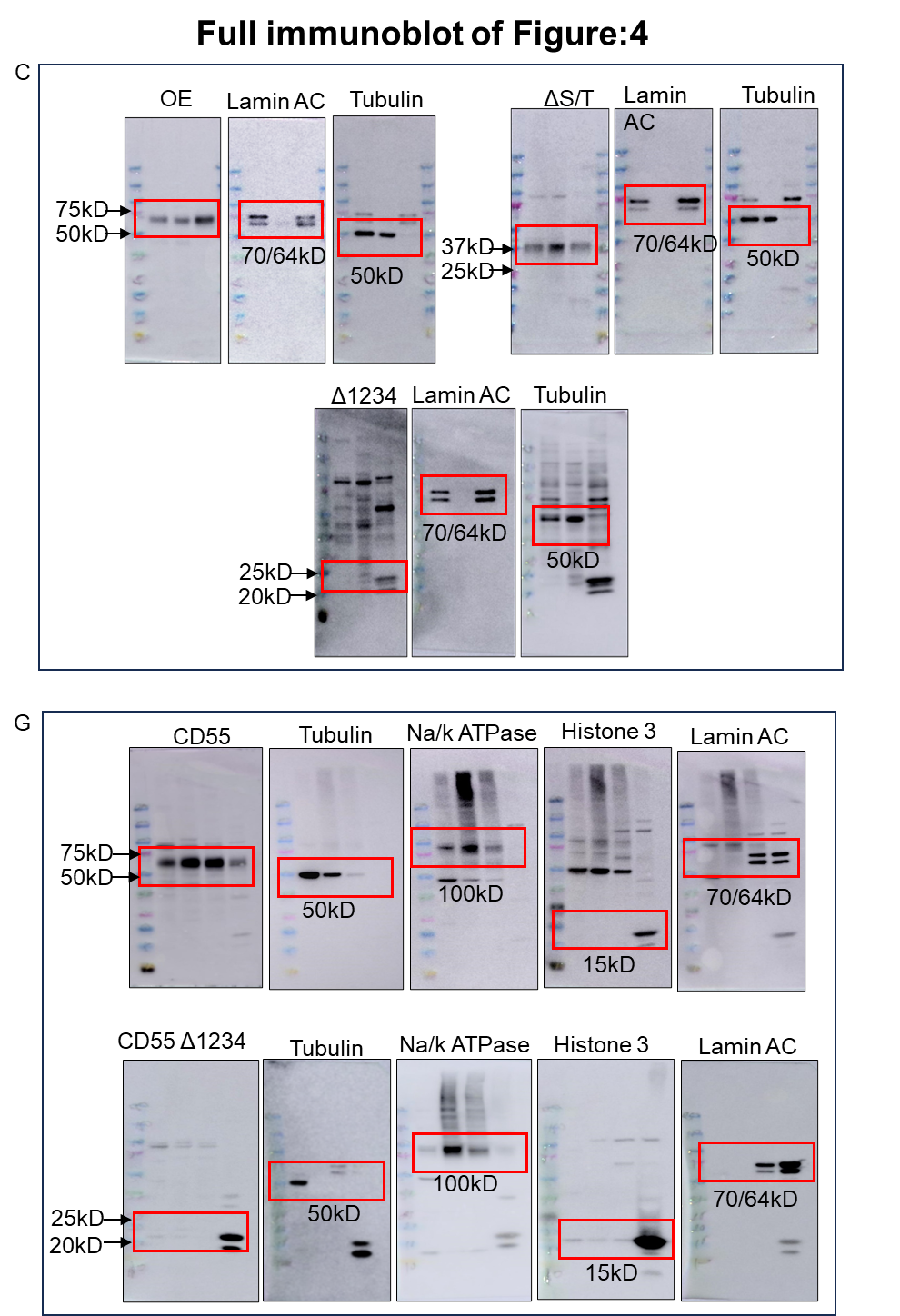


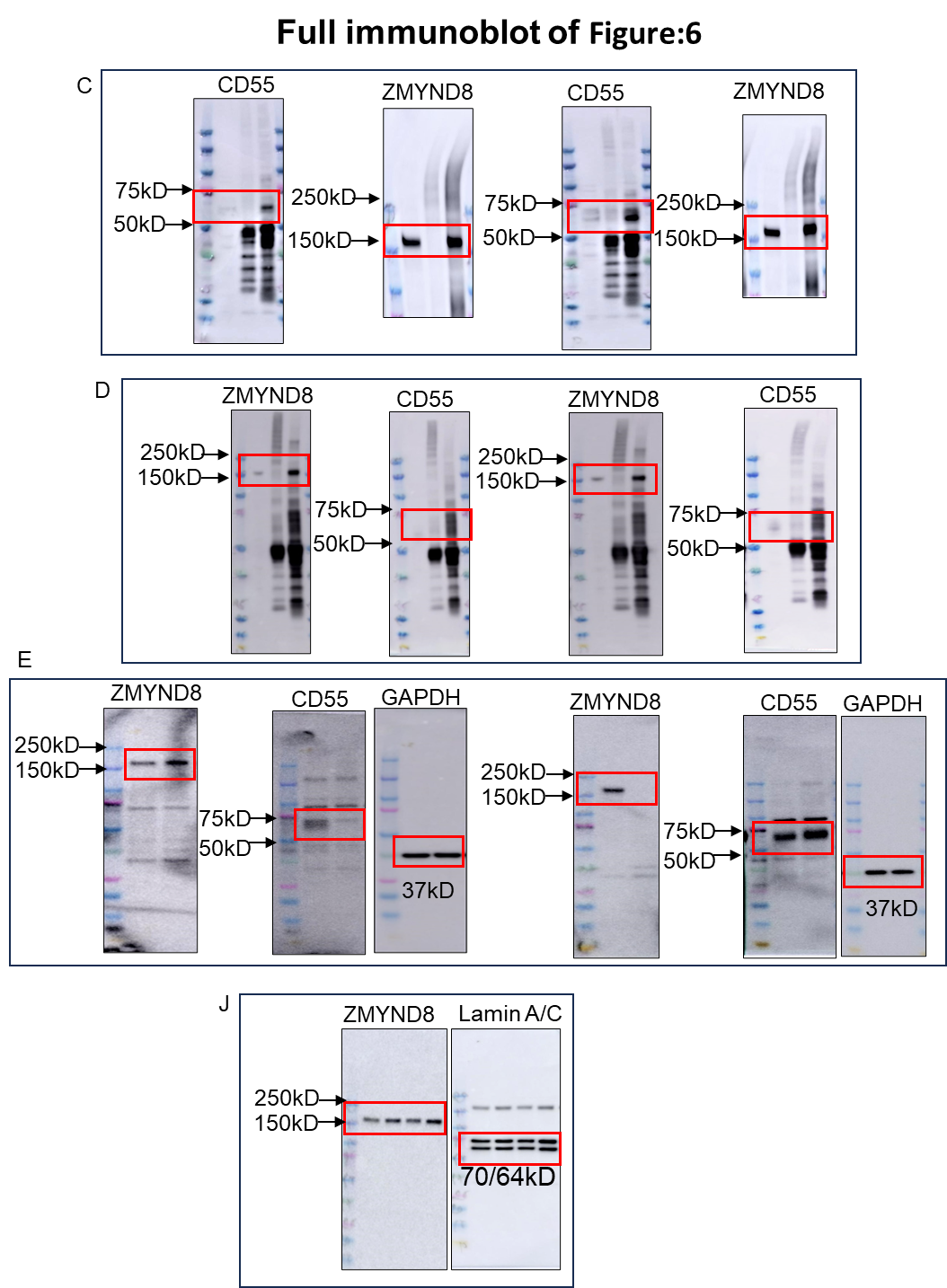


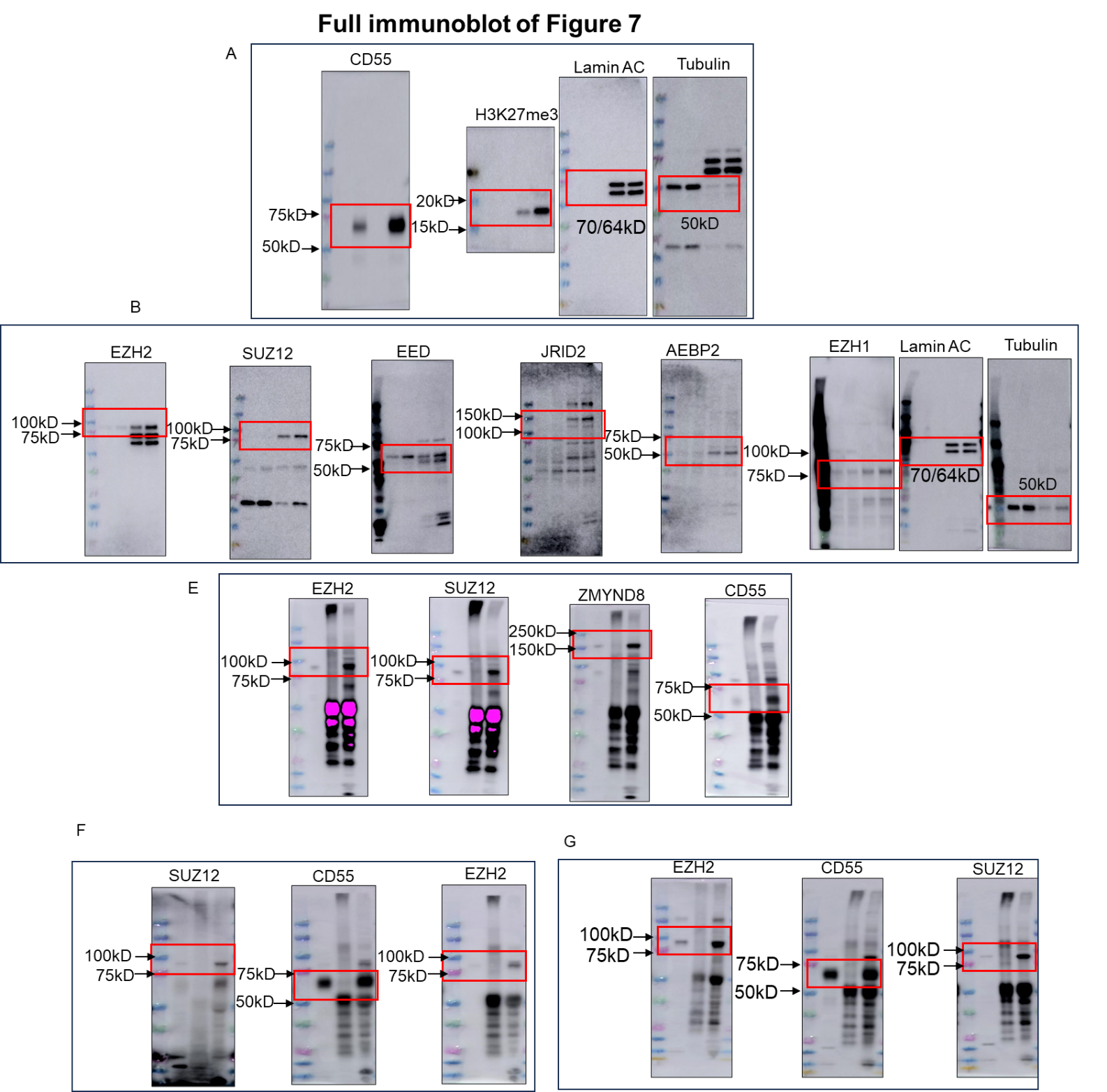


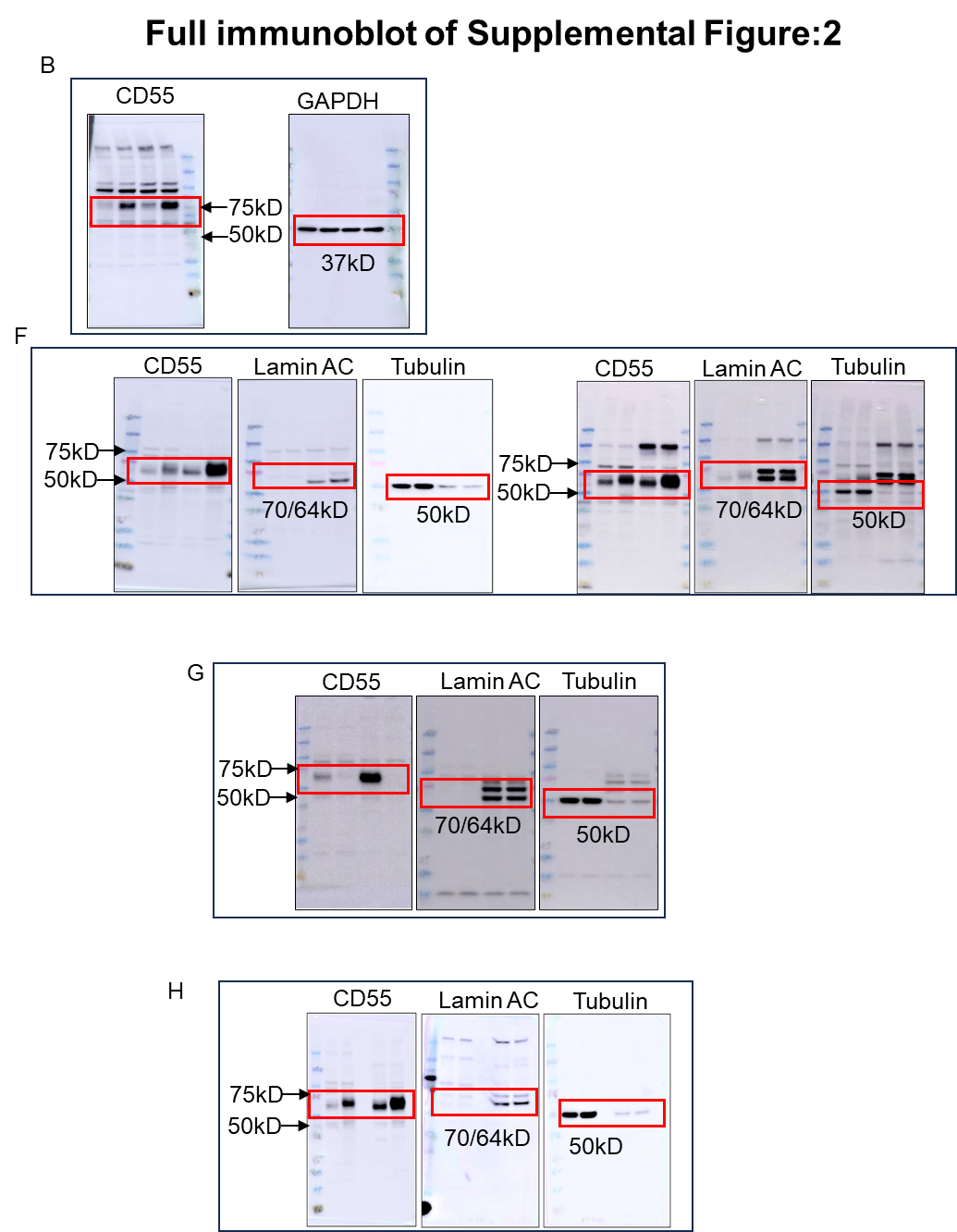


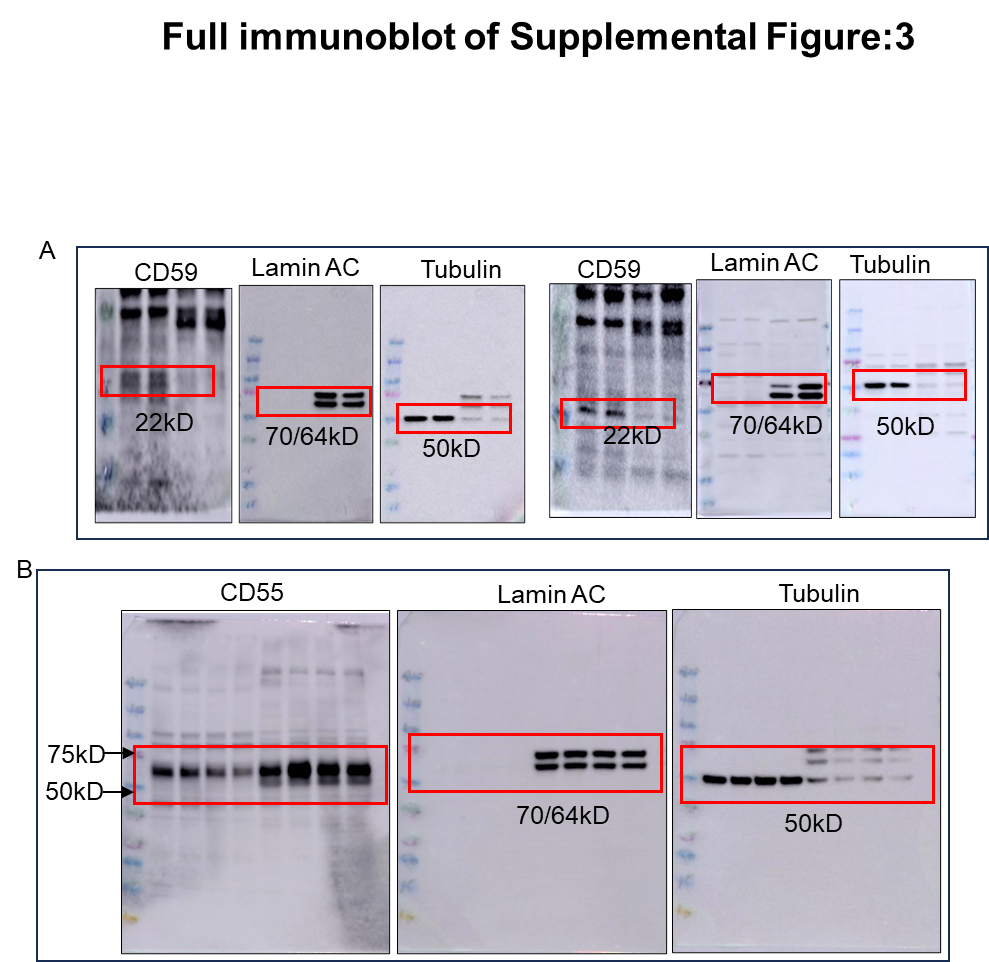


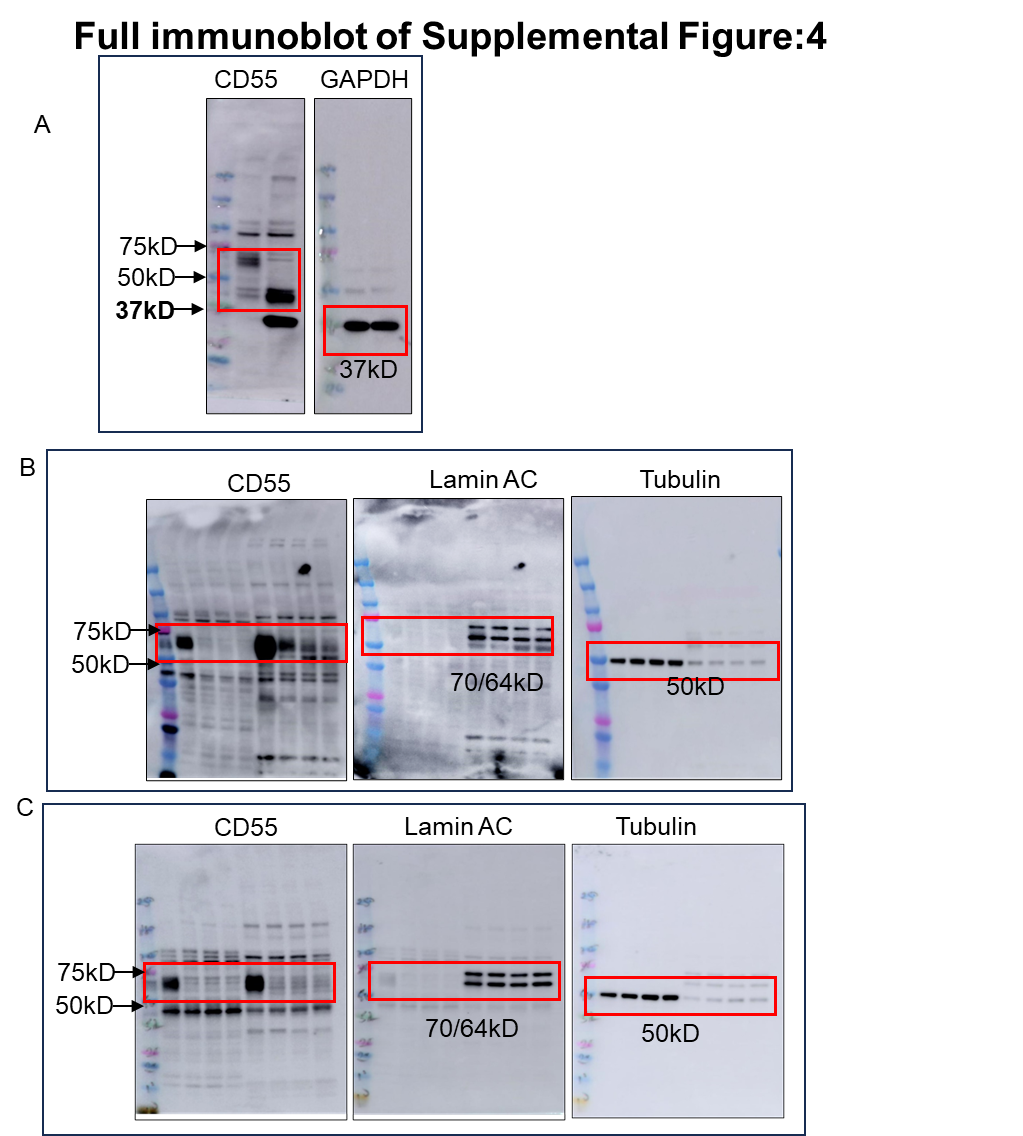


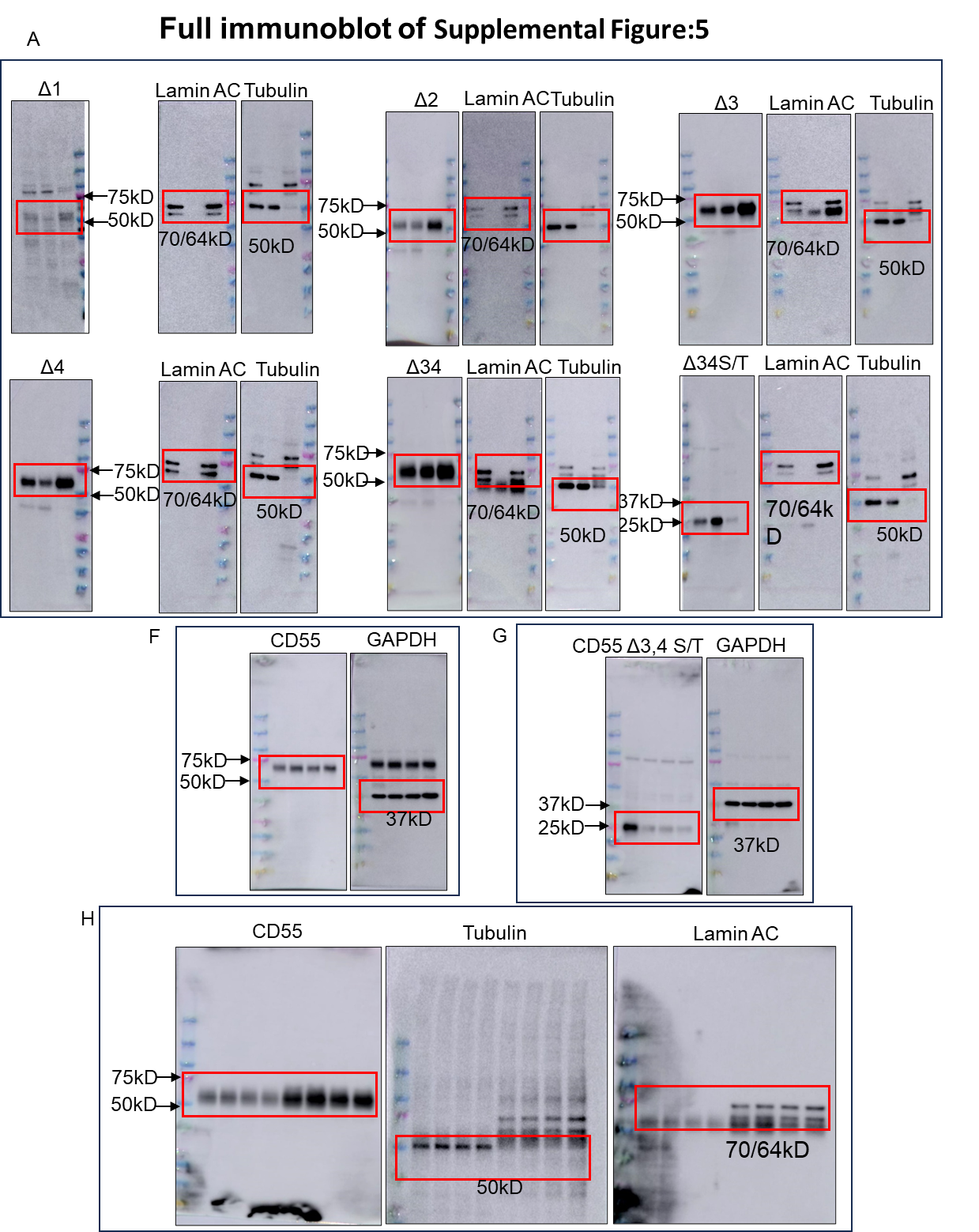


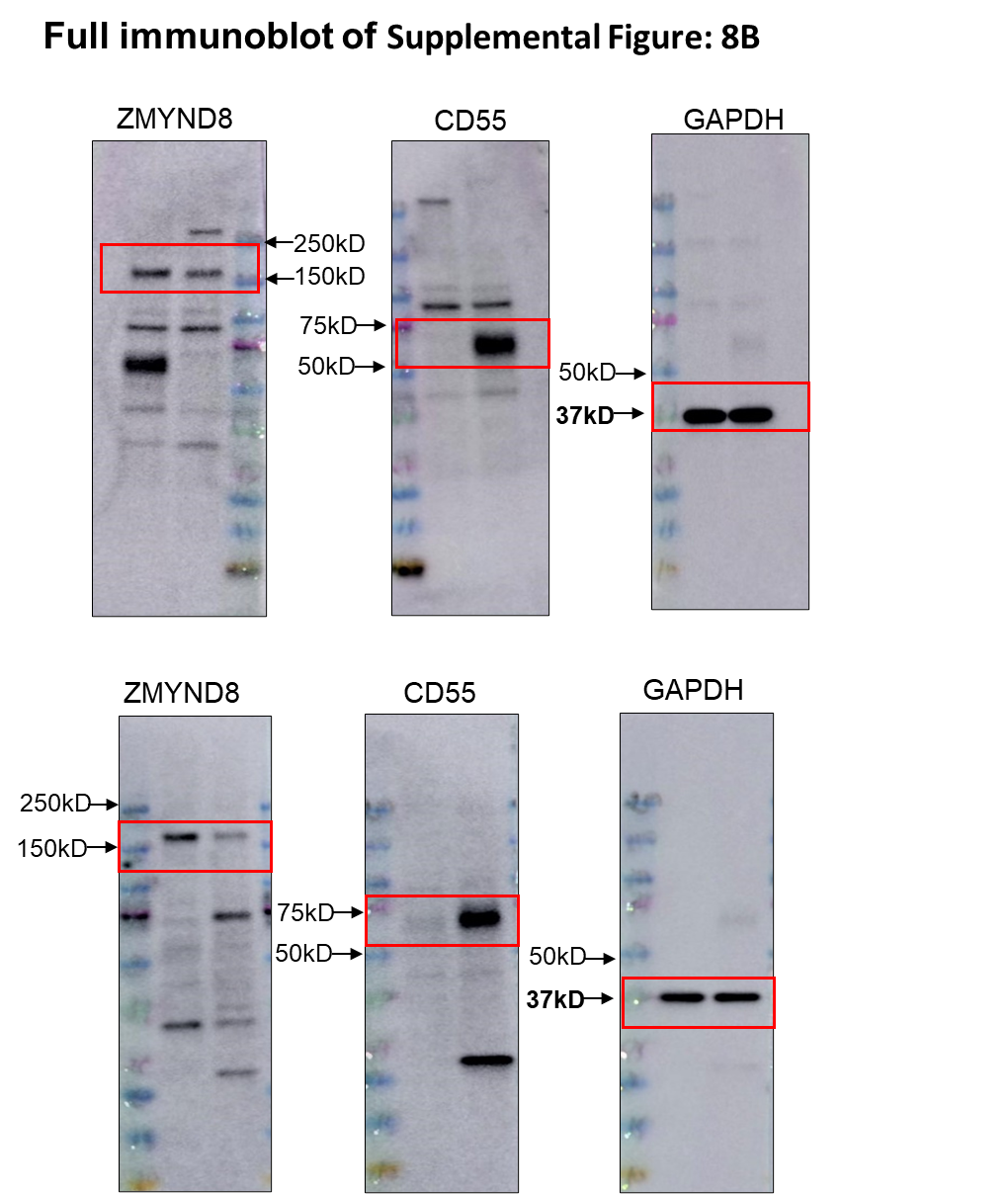

Supplement: Supplementary file 1 — Supplementary Material 1 [file 12943_2024_2028_MOESM1_ESM.docx]
